# Kinematic Responses to Water Treadmill Exercise When Used Regularly within a Sport Horse Training Programme: A Longitudinal, Observational Study

**DOI:** 10.3390/ani14162393

**Published:** 2024-08-18

**Authors:** Kathryn Nankervis, Carolyne Tranquille, Jack Tacey, Isabeau Deckers, Russell MacKechnie-Guire, Vicki Walker, Emily Hopkins, Richard Newton, Rachel Murray

**Affiliations:** 1 Equine Department, Hartpury University, Gloucester GL19 3BE, UK; carolyne.tranquille@outlook.com (C.T.); isabeau.deckers2@hartpury.ac.uk (I.D.); victoria.walker@hartpury.ac.uk (V.W.); 2 JBT Veterinary Physiotherapy, Nottingham NG10 4EH, UK; jttacey@hotmail.co.uk; 3 Centaur Biomechanics, Moreton Morrell, Warwickshire CV35 9BD, UK; russell@centaurbiomechanics.co.uk; 4 Regain Veterinary Physiotherapy, Bury St Edmunds IP31 3SL, UK; eghopkins@outlook.com; 5 Department of Medicine, University of Cambridge, Cambridge CB3 0ES, UK; rn428@cam.ac.uk; 6 VetCT, Cambridge CB3 0FA, UK; rachel.murray@vet-ct.com

**Keywords:** water treadmill, hydrotherapy, training, kinematics, exercise, sport horse

## Abstract

**Simple Summary:**

Water treadmill (WT) exercise is popular for cross-training of sport horses. The nature of the horse’s adaptation to WT exercise could be influenced by the type of session used and how often it is conducted. The study’s aim was to compare the gait characteristics of 48 sport horses during a standardised WT exercise test (WT_SET_) at the start, i.e., ‘week 0’, and after 20 and 40 weeks of WT exercise incorporated within their normal training programme. Horses were recruited from the existing client populations of two commercial water treadmill venues for the purpose of this longitudinal, observational study. Measurements of limb, back, poll, wither, and pelvic movement were taken during the WT_SET_ at weeks 0, 20, and 40. Compared to week 0, the results showed changes in gait characteristics during the WT_SET_ at weeks 20 and 40 that reflected adaptation to walking in water, including increased protraction and decreased retraction of the fore and hind limbs. Kinematic responses were primarily dependent upon water depth, but previous experience of the horses with WT exercise, the number of WT exercise sessions carried out per month, and the type of the WT exercise session used during training with respect to speed and depth used were also influential. The WT session design and frequency of use can influence the nature of the kinematic responses to water walking seen over time, suggesting that WT exercise sessions within a normal sport horse training programme could be designed in accordance with specific training goals.

**Abstract:**

Repeated exposure to water treadmill (WT) exercise could elicit kinematic responses reflecting adaptation to WT exercise. The study’s aim was to compare the responses of a group of sport horses to a standardised WT exercise test (WT_SET_) carried out at three time points, week 0 (*n* = 48), week 20 (*n* = 38), and week 40 (*n* = 29), throughout a normal training programme incorporating WT exercise. Horses were recruited from the existing client populations of two commercial water treadmill venues for the purpose of this longitudinal, observational study. Limb, back, poll, wither, and pelvic kinematics were measured during the WT_SET_ using videography, optical motion capture, and inertial motion sensors. Forelimb and hindlimb protraction increased (*p* < 0.001 for both), and forelimb and hindlimb retraction decreased (*p* < 0.001 for both) at week 40 compared to week 0. Caudal thoracic flexion–extension and lateral bend ranges of movement were greater at week 40 compared to week 0 (*p* < 0.001 and *p* = 0.009, respectively). Increased training speed was associated with increased craniocaudal poll movement (*p* = 0.021), decreased forelimb protraction (*p* = 0.008), and increased forelimb retraction (*p* = 0.021). In addition to characteristic changes in kinematics due to increasing water depth, regular WT exercise resulted in kinematic adaptation to movement in water. Factors such as the frequency of WT sessions and the type of session used with respect to depth and speed were seen to influence the nature of the adaptation. The results suggest that WT exercise sessions could be designed in accordance with specific training goals when used within a normal sport horse training programme.

## 1. Introduction

Water treadmill (WT) exercise is a popular tool for training and rehabilitation of horses. A wide variety of applications are described within the scientific literature, including sport horse training [1] and racehorse rehabilitation [2]. WT exercise exposes horses to different forces and biomechanical challenges compared to overground walking or walking on a dry treadmill belt. The volume of water displaced by the horse creates a buoyant force which assists with movement of the limbs in the vertical plane, whilst drag opposes forward movement of limbs. In terms of energetic demand, walking in water up to the level of the stifle requires greater oxygen uptake than walking on a dry belt [3], eliciting a conditioning response when applied within an 18-day conditioning programme [4]. Whilst all evidence suggests that WT exercise is a low-intensity, aerobic exercise [3,4,5,6,7,8,9], it is associated with significant kinematic adaptation compared with walking on a dry treadmill. WT exercise has been associated with increased distal limb flexion [10,11,12], increased stride length and reduced stride frequency [11,13,14], increased hindlimb protraction–retraction range of movement (ROM) [15], increased pelvic flexion and pelvic vertical displacement [16], and increased flexion–extension of the thoracolumbar spine ROM [17] when compared with walking on a dry treadmill.

Theoretically, repeated exposure to WT exercise and, hence, repeated performance of the characteristic movement patterns associated with WT exercise could be expected to elicit neuromuscular adaptation to WT exercise. Using accelerometers on the sternum and the sacrum [18], it has been demonstrated that increasing water depth and speed creates a shift towards more dorsoventral displacement and dorsoventral power, i.e., more up and down movement of the centre of gravity and relatively less longitudinal and mediolateral displacement. The authors suggested that if these modifications were maintained during overground locomotion, there would be evidence to recommend WT exercise for training sport horses since large dorsoventral displacement of the trunk in both walking and trotting has been associated with higher dressage scores in young horses [19]. Increased hindlimb protraction–retraction ROM [15,20] and increased lumbosacral flexion [16] and lumbar flexion [17], as seen even in relatively low water (i.e., below tarsal depth), could be hypothesised to cause adaptation of hindlimb and epaxial musculature when WT is used regularly. There is some evidence to support the use of WT training programmes for the development of hindlimb musculature, as both subjective visual assessment [21] and ultrasound measurement [22] have shown increased hindlimb muscle mass following twenty weeks and eight weeks of WT training, respectively. In the latter study, young Friesian naïve horses exposed to 5 days/week WT training at water depth mid-metacarpus and speed 1.25 m/s showed superior hindquarter muscle diameter compared with a dry treadmill-trained group. Another recent study [23] reported increases in back muscle profile (measured with a flexicurve) in six horses following four weeks of inclined WT exercise. It should be noted, however, that previous studies have found no evidence of any improvement in the oxidative or glycolytic capacity of the gluteal muscle following either four weeks [7] or eight weeks of WT training [8]. Based on the existing evidence, it would appear that the type of WT training undertaken in terms of water depth, speed, frequency of application, and duration of exposure could have a substantial influence on the locomotory response, and over time, the neuromuscular adaptation to it.

Physical properties of water are understood to have benefits that support the use of WT exercise for horses with pathology. This was demonstrated with an eight-week WT training programme (five days per week for a maximum of twenty minutes at shoulder depth) in horses recovering from experimentally induced carpal joint osteoarthritis [24]. In this study, there were improvements in balance control assessed by postural sway analysis on a force platform in the WT-trained horses. Horses exercised on the WT showed decreased craniocaudal and mediolateral sway compared with the control group. The authors suggested that the WT training programme may have increased afferent excitation of the motor neuron pool for the muscles responsible for stabilizing the thoracic and pelvic limbs. In addition, there were increases in passive carpal joint ROM measured using goniometry. However, a shorter (10 days) training period in healthy horses judged free from lameness did not result in any changes in overground forelimb kinematics [11]. It remains to be seen whether regular WT exercise over longer time periods is associated with changes in overground kinematics.

All the aforementioned studies used WT exercise as the primary means of exercise for the duration of the study. This may be typical of the way the modality is used within some rehabilitation programmes, but for normal, healthy sport horses, WT exercise is usually just one type of exercise within a varied training programme [1]. With repeated WT exercise, horses could be expected to develop neuromuscular pathways that reflect kinematic adaptation to movement in water. Training adaptations vary according to the frequency, duration and intensity of the applied load [25]. The aim of this study was to evaluate the responses to WT exercise of a group of sport horses that all had prior experience with WT exercise and carried out WT exercise regularly as part of their normal training programme. The objective was to measure the kinematic responses of a group of horses to a standardised WT exercise test (a WT_SET_) at three time points over a 40-week period: 0 weeks, 20 weeks, and 40 weeks, and to compare the responses over time. It was hypothesised that, in addition to the effects of water depth and speed, the kinematic responses to the WT_SET_ would change with time and would be influenced by the prior experience of the horses to WT exercise, the frequency of exposure to WT exercise during training, and the nature of the WT exercise experienced during training with respect to the speed and depth used. The findings of the study could be applied by practitioners designing individual WT exercise sessions and training programmes incorporating WT exercise.

## 2. Materials and Methods

The study was approved by the Ethical Review Committee of the Animal Health Trust (project number: 42-2017).

### 2.1. Horses

A sample of 48 sport horses accustomed to WT exercise (aged 11.3 ± 3.4 years) between 148 cm and 188 cm in height were used. Fifty-eight percent of the horses were Warmblood, 27% were TB/TB cross, and 15% were ‘other’ breeds. Horses took part in the following activities: dressage (*n* = 29), show jumping (*n* = 5), eventing (*n* = 11), and general purpose (*n* = 3). Across the disciplines, there were 17 horses that could be considered ‘elite’, i.e., competing in dressage at advanced-medium or above, competing in showjumping at Grade A or above, or competing in eventing at intermediate or above. There were 26 non-elite and 5 non-competing leisure horses. A power calculation based on data from a previous study [12] indicated that a minimum of 16 horses would be required to detect a difference at a significance level of *p* ≤ 0.05 and 90% study power. This calculation was based on peak metacarpophalangeal flexion angles measured in dry conditions (175° ± 13) and with water at carpal depth (154° ± 16) during WT exercise conducted at 5.7 km/h.

Horses were recruited from two commercial WT venues with large client bases within the South of England. Horses were included if they used the WT regularly (weekly *n* = 20, fortnightly *n* = 19, three times per month *n* = 4, more than once a week *n* = 5), were not under any investigations relating to orthopaedic disease or loss of performance, and were considered sound by their owners. This was verified by a gait evaluation based on an International Equestrian Federation pre-competition veterinary assessment by an orthopaedic specialist (RCM), including straight-line walk and trot assessment at weeks 0, 20, and 40. Horses were excluded if they were given a lameness score of 1/10 or greater [26] on a straight-line trot up on a firm surface and/or demonstrated reactivity to back palpation at weeks 0, 20, and 40.

### 2.2. Training Programmes

Week ‘0’ was between Feb 2019 and May 2019, week ‘20’ between July 2019 and September 2019, and week 40 between Dec 2019 and February 2020. Over the training period, all horses continued with their normal training and competitive programmes as designed by the owners, riders, and trainers. The WT training programme was defined on an individual basis by the WT venue and operators and discussed with the research team. The horses had differing levels of prior experience at week ‘0’ and were grouped and defined as follows: group one: less than 5 weeks’ experience (*n* = 2), group two: 5 to 24 weeks (*n* = 9), group three: 25 to 52 weeks (*n* = 16), group four: 53 to 78 weeks (*n* = 6), group five: 79 to 104 (*n* = 11) weeks, and group six: more than 104 (*n* = 5) weeks’ experience.

Horses were exercised according to guidelines for WT exercise in healthy horses [27], as available on British Equestrian’s website. Training sessions were no more than 25 min in duration, at a training speed of 3.6 kph to 5.4 kph (mode 4.6 kph), i.e., 1.0–1.5 m/s (mode 1.3 m/s), and horses carried out their WT exercise sessions for the purpose of training at the following water depths: fetlock (*n* = 4), midcannon (*n* = 16), distal carpus (*n* = 5), and carpus (*n* = 23).

### 2.3. Testing Protocol at Weeks 0, 20 and 40

**Figure 1:**
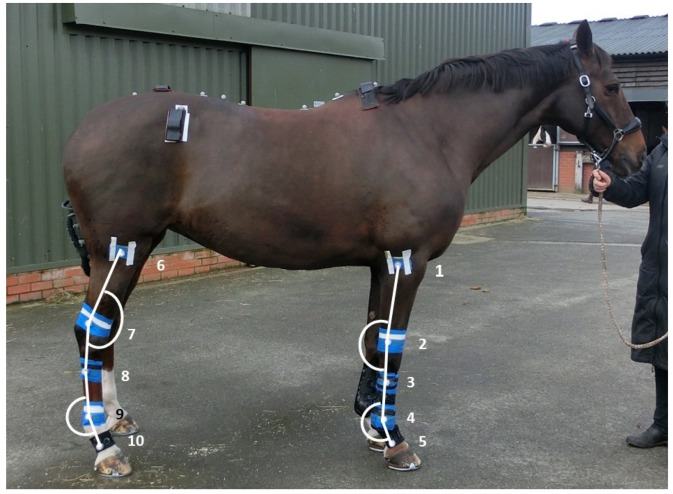
Marker and sensor placement for data collection and method of carpal, metacarpophalangeal, tarsal, and metatarsophalangeal joint angle measurement.

All horses were fitted with 30 mm diameter, waterproof light-emitting diodes (LEDs) affixed to pre-determined locations with wraps (Figure 1) to measure joint angles using videography at 240 Hz (Casio EX-FH250, 4 m field of view). The cameras were mounted on a tripod positioned parallel to the WT treadmill, with one camera capturing the forelimb and the second capturing the hindlimb. LEDs were attached over the following anatomical locations: 1. lateral epicondyle of the humerus, 2. lateral styloid process of the ulna, 3. lateral proximal aspect of the third metacarpus, 4. lateral distal aspect of the third metacarpus, 5. lateral aspect of the mid proximal phalanx of the forelimb, 6. head of the fibula, 7. lateral malleolus of the fibula, 8. lateral proximal aspect of the third metatarsus, 9. lateral distal aspect of the third metatarsus, and 10. lateral aspect of the mid proximal phalanx of the hind limb. Water quality was optimal on test days to ensure marker visibility throughout data collection.

Five MTw inertial measurement units (60 Hz) (IMU) (Xsens Technologies B.V. Enschede, The Netherlands) for use within a validated sensor-based system (EquiGait Ltd., Hertfordshire, UK) [28,29] were attached to the poll, withers, sacrum, and left and right tubera coxae (LTC and RTC). Horses were fitted with reflective back markers (19 mm diameter hemispheres placed on the sixth, tenth, thirteenth, and eighteenth thoracic; third and fifth lumbar; and third sacral vertebrae (T6, T10, T13, T18, L3, L5, and S3, respectively). Back markers were tracked using six Miqus motion capture cameras (240 Hz, Qualisys Medical AB, Göteborg, Sweden) positioned around the WT. Cameras were calibrated with a resolution of ~1 mm. All markers and sensors were secured prior to exercise and remained in place throughout the duration of the testing.

Horses performed the 19 min WT_SET_ on the WT (Activomed, FMBs), which included three minutes of filling time. The same model of treadmill was used in both venues. Horses wore bridles or leather headcollars and were held by handlers on both the left and right side to ensure they were straight and maintained a central position on the belt. Data were collected from the right side of the WT. An experienced WT operator on the left-hand side of the horse was responsible for identifying and confirming the water depth.

The same WT_SET_ with incremental increases in water depth at a set speed was carried out by all horses. The highest depth (distal radius) was greater than that used in training (carpus), and the test speeds were slightly higher than those experienced in training. The WT_SET_ was thus designed to be more challenging than the training session, but not so challenging it could not be completed by all horses. Horses walked at the same speed for each test condition at weeks 0, 20, and 40. The test speed for each horse was determined by the WT operator and ranged from 4.5 to 6.2 kph (mode 5.4 kph), i.e., 1.3–1.7 m/s (mode 1.5 m/s) between horses. This was selected based on visual observation of gait quality [27,30], but was kept the same for each horse over the three time points. Data were captured with the horses walking in the following conditions: (1) dry belt, and then water depth at the level of each of the following anatomical reference points; (2) coronary band; (3) lateral aspect of the metacarpophalangeal joint; (4) proximal metacarpal; and (5) distal radius. Each test condition lasted three minutes, with data being collected only after the first 30 s at each depth to allow acclimation to each new depth. The operator understood that they could stop the test at any point if necessary based on an individual horse’s response and/or safety reasons.

Limb kinematics were quantified using videography, and upper body displacements were measured using IMUs. In addition, motion capture tracked displacements of back markers for the purpose of measuring thoracolumbar ROMs during the WT_SET_. All videographic, IMU, and motion capture data were manually synchronised and collected for a minimum of 20 s at each water depth for each horse. Where stride frequency was low during the highest depths of the WT_SET_, two sets of 20 s data collection were carried out to achieve at least 25 strides in order to enable automatic processing of IMU data within the Equigait software version 4 (EquiGait Ltd., Hertfordshire, UK.)

### 2.4. Data Analysis

#### 2.4.1. Limb Kinematics

Carpal, metacarpophalangeal joint (MCPJ), tarsal and metatarsophalangeal joint (MTPJ) angles were measured at peak flexion during swing (i.e., the smallest carpal/tarsal angle during the swing) using the palmar and plantar aspects of the limb. Peak protraction and reaction were measured when the limb was maximally extended cranially/caudally. All measurements were acquired using digital image analysis software (Pro Analyst, Professional Edition, Xcitex, Woburn, MA, USA). Markers were tracked manually. The repeatability of marker tracking had been previously determined by tracking all markers and deriving angles five times in three horses. A coefficient of variance of <3% was calculated and deemed acceptable [12].

#### 2.4.2. Dorsal Midline and Pelvic Displacements

Dorsoventral, craniocaudal, and mediolateral ROM for the poll, withers, LTC and RTC, and sacrum were measured for a minimum of 25 strides for each horse in each condition. Videography, motion capture, and IMU systems were manually synchronised.

#### 2.4.3. Thoracolumbar Flexion–Extension and Lateral Bending Ranges of Motion

Flexion–extension (FE) and lateral bend (LB) ROMs of the thoracolumbar spine were calculated using the method of [31] following stride splitting (using pelvic markers) and filtering using a low-pass, fourth-order Butterworth filter with a cut-off frequency of 10 Hz within MATLAB version 2019a (The Mathworks, Natick, MA, USA).

### 2.5. Statistical Analysis

Descriptive statistics were carried out for each variable at each condition using statistical analysis software (JASP). Univariable mixed-effects linear regression analyses were used to examine the relationship between outcome variables; then, mixed-effect multiple linear regression models containing limb angles, dorsal midline and pelvic displacements, and thoracolumbar FE and LB ROM as outcome variables and each predictor variable frequency of WT exercise: training speed, training WT depth, prior WT experience, frequency of WT exercise, discipline, competition level, testing week (0, 20 and 40), water depth, and test speed, with horse identity used as a random effect term in order to control for the multiple “clustered” measures that were taken from each horse (Stata 15.0). All outcome variables were checked for normality of distribution and were considered appropriate for this form of analysis. A significance value of *p* ≤ 0.05 was applied in all analyses.

## 3. Results

A total of 38 horses completed week 20, and 29 horses completed week 40. Between 0 and 20 weeks, 10 horses were lost for reasons not related to the study (unavailable to attend testing (*n* = 5), non-WT-related skin disease (*n* = 2), lameness (*n* = 3)). Between week 20 and 40, a further nine horses were lost (could not attend testing (*n* = 6), lameness (*n* = 3)). All horses successfully completed the WT_SET_, although two horses did not complete the proximal metacarpal and distal radius steps of the WT_SET_ at week 0, but then completed the full test by weeks 20 and 40.

### 3.1. Limb Kinematics

The significant results of the multivariate model for limb kinematic data are shown in Table 1. Water depth was a significant predictor of all limb kinematic variables within the model and was associated with the highest regression coefficients. The only exception to this was ‘training at carpal depth’ as a predictor for the outcome variable, ‘hindlimb protraction’. Non-significant findings can be found in Supplementary Table S1.

#### 3.1.1. Forelimbs

**Figure 2:**
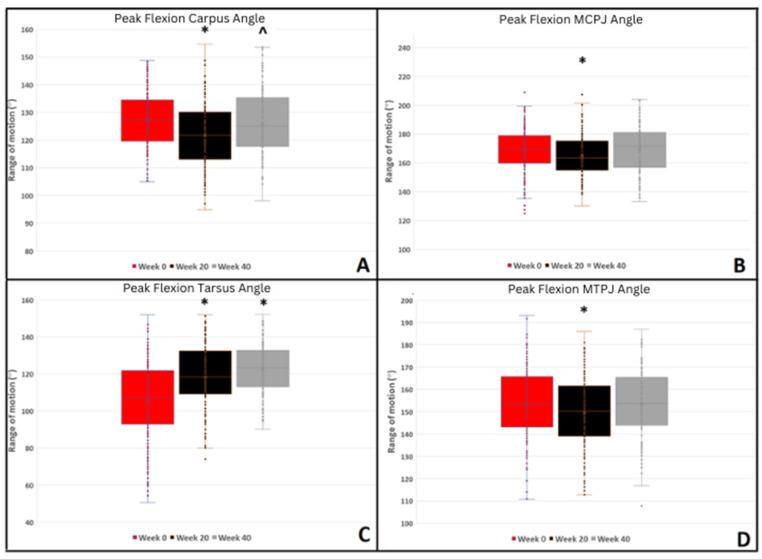
Summary of peak flexion of fore and hindlimb joint angles during the water treadmill test for all water depths pooled for 0, 20, and 40 weeks. Significant differences are denoted by symbols. * = *p* ≤ 0.001, ^ = *p* ≤ 0.010.

**Figure 3:**
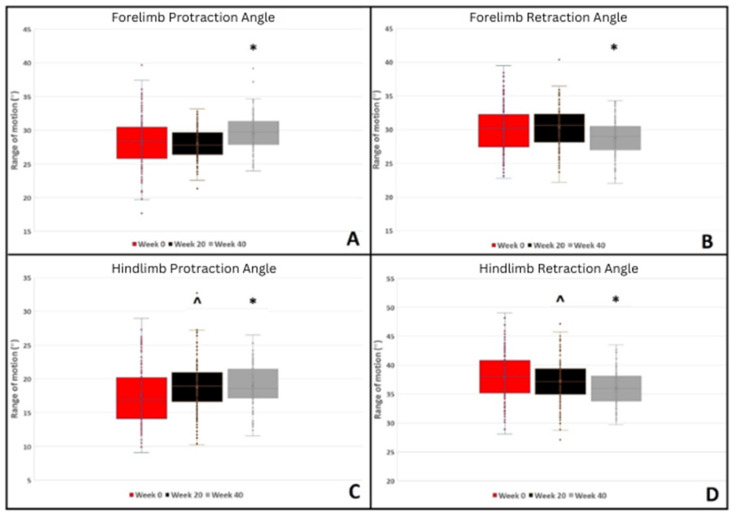
Summary of maximum fore and hindlimb protraction and retraction angles during the water treadmill test for all water depths pooled for 0, 20, and 40 weeks. Significant differences are denoted by symbols. * = *p* ≤ 0.001, ^ = *p* ≤ 0.010.

Peak flexion of the MCPJ, carpal, MTPJ, and tarsal joints increased with water depth during the WT_SET_. Peak flexion of the MCPJ increased at week 20 compared to week 0, whilst carpal flexion increased at week 20 and week 40 compared to week 0 (Figure 2). Forelimb protraction increased, whilst retraction decreased at week 40 compared to week 0 (Figure 3). Increased WT_SET_ speed was associated with reduced peak carpal flexion whilst ‘increased experience of WT exercise prior to week 0’ was associated with increased peak carpal flexion. Increased training speed was associated with decreased forelimb protraction and increased forelimb retraction.

#### 3.1.2. Hindlimbs

In the hindlimb, peak MTPJ flexion increased at week 20, whilst tarsal flexion decreased at weeks 20 and 40 compared to week 0 (Figure 2). In the hindlimb, increased protraction and decreased retraction were observed at both 20 and 40 weeks compared to baseline (Figure 3).

### 3.2. Dorsal Midline and Pelvic Displacements

A summary of the significant results of the multivariate model for dorsal midline and pelvic displacement data can be seen in Table 2. As for limb kinematics, water depth during the WT_SET_ had more influence on outcome variables (based on regression coefficients) than most other predictor variables, with water depth increasing dorsoventral movement of the dorsal midline and pelvic landmarks. Craniocaudal movement of the poll was more sensitive to water depth increases than either dorsoventral or mediolateral poll movement and was decreased at week 20 and week 40 compared with week 0, but increased with training speed. Craniocaudal movements of all other dorsal midline and pelvic landmarks were, in contrast, much less influenced by water depth. Mediolateral displacement of the poll increased at week 40, and mediolateral movement of the withers increased at week 20 and 40 compared to week 0. A higher frequency of WT exercise reduced mediolateral movement at the wither to the same degree as increasing water depth. Dorsoventral displacement of the sacrum and LTC was reduced at week 20 compared to baseline, but no changes were seen in RTC, although mediolateral displacement of RTC increased at week 20 and week 40. Non-significant results can be found in Supplementary Table S2.

### 3.3. Flexion–Extension and Lateral Bend Range of Movement of the Back

Table 3 summarises the significant results of the multivariable analyses for FE and LB ROM of the thoracolumbar spine. FE ROM for T10 to L5 increased with increasing water depth. LB ROM increased with water depth at T10, T13, T18, and L3 and reduced at L5. In contrast, the frequency of use decreased LB ROM at T10. Horses using the WT for between 5 and 24 weeks at time ‘0’ (Group 2, *n* = 9) had greater increases in FE ROM at T10 in water at proximal carpal depth (compared to dry belt) than horses with fewer than 5 weeks experience, and horses using the WT for between 5 and 24 weeks had a lower LB ROM at T10. LB ROM at T13 increased with the test speed. Only one anatomical location was influenced by week (i.e., training); T18 FE and LB ROM increased at week 20 and week 40 compared to the baseline. The training depth of the midcannon was associated with greater T18 and L5 LB ROM compared to fetlock depth. However, 53–78 weeks of experience with WT exercise at week 0 reduced LB ROM at T18 compared to horses trained for fewer than 5 weeks. Non-significant findings can be found in Supplementary Table S3.

## 4. Discussion

In accordance with our hypotheses, factors such as time point (i.e., week 0, week 20, and week 40), the prior experience of horses to WT exercise, and the frequency and nature of WT exercise experienced during training influenced the limb, dorsal midline, pelvic and back kinematics during the WT_SET_. The addition of water during the WT_SET_ increased distal limb joint flexion compared with dry (baseline) conditions, as expected and seen previously [10,11,12,30], but the results showed additional responses reflecting changes over time. Regular WT exercise within a normal sport horse training programme resulted in increased flexion during swing of the metacarpophalangeal and carpal joints within the WT_SET_ at 20 and 40 weeks, an increase in forelimb protraction, and a decrease in forelimb retraction. The kinematic responses of the forelimb could indicate adaptation to walking in water, brought about by an increased ability to flex and elevate the distal limb joints and by an increased ability to protract the forelimb against increased drag. Previously, a period of WT training was seen to restore carpal joint flexion in horses with compromised ROM following surgically induced carpal osteoarthritis [32]. The improved ROM seen in WT exercise horses compared to the control group was thought to be due to the physical properties of water leading to a reduction in joint inflammation. Whilst the same outcome was seen during the WT_SET_ in this study, the mechanism was likely to be different in these healthy horses exercising in lower water. Carpal joint flexion was decreased (i.e., the carpal angle increased) by increasing test speed (3.28° per m/s), which is in contrast to an increase in carpal flexion of similar magnitude (3.24° per m/s) seen previously in a group of Thoroughbred horses exercising at different speeds in water up to the level of the carpus [31].

During the WT_SET_, forelimb protraction was greater at coronary band depth compared with the dry belt, but forelimb protraction was otherwise unaffected by depth, as has been seen previously in relatively low (30 cm) water [20]. Forelimb protraction during WT walking in high water (stifle depth) has been seen to be lower than that during walking on a dry treadmill belt [15], suggesting a limitation of forelimb protraction in high water. McCrae et al., 2021 [11] observed some reduction in shoulder extension when walking in high water (stifle depth). Increased forelimb muscle activity during a single WT session suggests that increased muscular work is required in order to extend and protract the forelimb against the resistance posed by the water [33], and increases in the diameter of the forelimb muscles (cervical and thoracic trapezius, brachiocephalicus, triceps brachii, and biceps brachii) seen following 8 weeks of WT training in comparison to dry treadmill training suggests adaptations that support distal limb elevation and protraction [22]. Some adaptation of forelimb kinematics has already been observed in a shorter period of WT training in which the belt speed was gradually increased [11]. McCrae et al. 2021 [11] found a decrease in shoulder extension in stifle-depth water and an increase in shoulder extension in carpal-depth water as a result of eight consecutive days of water treadmill training, which culminated in belt speeds of 1.3–1.5 m/s in stifle-depth water. In the present study, forelimb protraction was increased at week 40 compared to week 0. Although the increase was modest in real terms, there was a similar regression coefficient to the decrease in forelimb protraction associated with an increase in training speed of 1 m/s. Any potential improvement in forelimb protraction due to training could, in theory, be negated if high training speeds, particularly at higher depths, were used. The faster the limb is required to advance through the water, the greater the drag force opposing it.

Tarsal joint flexion increased with depth, as expected, but decreased with increased training time, i.e., decreased at weeks 20 and 40 compared to week 0. The training adaptation seen in the tarsal joints is, therefore, opposite to that of the carpal joint. The MTPJ, however, like the MCPJ, demonstrated increased flexion between weeks 0 and 20. As in the forelimb, increased water depth within the WT_SET_ was associated with increased hindlimb protraction and reduced hindlimb retraction. WT training may develop hip flexors and hypaxial musculature, enabling the hindlimb to be placed closer to the centre of mass on initial ground contact and hip extensor strength, increasing the power developed within retraction. This is supported by reports of increased hindquarter muscle mass following WT training [21,22]. Increased hindquarter muscle mass following WT training also explains why horses with more than 5 weeks’ experience with WT exercise at the start of the study show less MTPJ flexion than less experienced horses, concurrent with a shift towards a reduced need to minimize drag by means of flexion of distal joints, leading towards an improved ability to oppose drag.

During the WT_SET_, there was an increase in dorsoventral and mediolateral displacement (the latter to a lesser degree) of all the dorsal landmarks—poll, wither, sacrum, LTC, and RTC—with water depth. Increased dorsoventral displacement of the spine with water depth has been seen in previous studies [12,31,34]. During the WT_SET_, only the poll had appreciable craniocaudal movement (i.e., >10 mm), which increased with depth to a greater extent than dorsoventral or mediolateral displacement, as evidenced by the relative regression coefficients at each water depth. It was interesting that the craniocaudal movement of the poll was reduced with training but increased with training speed, as was forelimb protraction. A previous study of Thoroughbreds walking on a water treadmill found that craniocaudal movement of the poll increased with belt speed [31]. Guidelines suggest that excessive craniocaudal head and neck movement could indicate that the belt speed at any given depth is too high [28]. Perhaps higher WT training speeds result in a movement strategy that favours greater utilisation of extrinsic forelimb protractors, as indicated by the greater craniocaudal movement of the poll. The findings of this study support practice guidelines regarding avoidance of high training speeds.

Dorsoventral displacement of the withers, sacrum, and pelvis increased with water depth, with regression coefficients showing that the dorsoventral displacement of the sacrum was approximately 1 cm more than the withers at the highest depth. Increasing dorsoventral displacement of the trunk and hindquarters has been seen previously [18,31,33]. Dorsoventral displacement of the sacrum was greater in horses with over 5 weeks’ experience with WT exercise and was increased by a higher frequency of WT exposure. It is possible that this reflects the development of a ‘stepping over the water’ strategy within the early adaptation to the exercise and/or reflects the training depths used during habituation to treadmill exercise, which can be considerably lower than carpal depth [1]. Whilst the sensors are measuring displacement, not absolute position, when taken with the visual impression, there is a tendency for the hindquarters to be elevated more than the withers during certain parts of the stride cycle, which may contribute to a limited ability to flex the thoracic spine during water treadmill exercise [17]. This, coupled with increased mediolateral displacement of the sacrum and LTC and RTC with water depth, greater than that of the withers, could contribute to increased axial torque around the mid-caudal thoracic spine. Displacement of the withers both dorsoventrally and mediolaterally was decreased by the frequency of exposure of the horses to WT exercise, but not by ‘time’. Perhaps variables influenced by frequency reflect changes in technique, as opposed to absolute changes in muscle development/muscle strength. King et al., 2013 [24] found decreased postural sway, i.e., increased postural stability, in horses with surgically induced osteoarthritis when trained on a WT in shoulder-depth water five times per week in comparison to those trained on a dry treadmill. WT exercise at the relatively lower water depths used in our study requires the upper body to remain upright in the face of increased forelimb joint elevation [11] and decreased stance time [10,11], which may present the horse with novel postural and proprioceptive challenges and may also stimulate postural stability.

With increasing water depth, hindlimb protraction and retraction increased (retraction more so than protraction), as seen previously [15,20]. In a similar way to the response of the forelimb, the hindlimbs showed training responses that suggested improved ability to move the limb against the drag imposed by the water, in that hindlimb protraction increased and retraction decreased. Thus, 40 weeks of WT training moderated the extent to which the hindlimb ‘trails’ behind the horse by approximately one-third of the increase associated with increasing water depth from the baseline to the distal radius. In a previous study [31], craniocaudal movement of the poll was suggested to be a useful indicator of the degree of extension of the posture created by a trailing hind limb. In the current study, craniocaudal poll movement and hindlimb retraction were both reduced by training, reinforcing the use of these two variables as indicators of the quality of the response of the horse to WT exercise, where an increase suggests poor quality.

FE ROM of T10, T13, T18, L3, and L5 increased with water depth during the WT_SET_. Increases in FE ROM between T10 and L3 due to increased water depth have been seen previously [17,31]. Changes in LB ROM with depth varied according to spinal region. There were only very small increases in LB ROM at T10 with water depth, and T10 LB ROM was decreased by frequency of use, perhaps related again to increased stability of the forelimb, as also indicated by reduced mediolateral wither movement with increased frequency of exposure. The biggest increases in LB ROM were seen around T13 in proximal-metacarpal-depth water compared to baseline (dry belt). Changes in thoracolumbar back movement over time were largely unremarkable, except for an increase in FE ROM and LB ROM at T18, which reflected greater caudal thoracic mobility. Alterations in positioning of the hindlimb further towards the centre of mass (evidenced by increased protraction and decreased retraction) may have influenced the increases in both FE and LB ROM of T18, which were also seen with training. A recent study measuring epaxial muscle profiles at the start of and at each week throughout a 4-week inclined water treadmill training programme found the most significant increases at T18, but since there was no control group exercising on either a level WT or a dry treadmill, it is not clear whether inclination, the addition of water, or time were most influential in that case.

The aim of this study was to evaluate changes over time in a group of sport horses using WT exercise within their normal training programmes. By far the most influential factor within the WT_SET_ session was water depth. However, there were also smaller relative changes over time (0, 20, and 40 weeks) in certain variables, reflecting adaptations to walking on a WT when used just once every two weeks. Training speed was found to influence head (poll) displacement and forelimb protraction, whilst frequency of exposure influenced wither and pelvic displacements. Like any other element of training, WT exercise should be designed to support individual training goals. This study has shown that the design of an individual WT session and the nature of WT training undertaken can influence the horse’s responses to water walking, hopefully stimulating further specific investigation of these variables within future WT training studies. With more knowledge, we can begin to develop more informed training programmes, incorporating combinations of water depth, speed, duration, and frequency to best support training goals. However, the real test of efficacy of WT exercise within a training programme would be improvements in function, performance, and/or soundness over time, factors which were not measured in this study.

Lack of control of the riding training programmes and the individual WT training programmes is a key limitation of this study. This was deemed necessary in favour of obtaining a large representative sample. Ideally, all horses would have been naïve to WT exercise at time 0. The findings from the multivariate analysis within this study could assist in the design of future studies to address how different types of WT training may influence variables in horses without any prior experience with WT exercise. Evidence shows that horses can vary in their individual responses to WT walking, e.g., exhibiting different thresholds for peak tarsal flexion [12] and peak pelvic displacement [17], and in their ability to cope with increases in belt speed [31]. Hence, each venue was allowed to design the programmes in line with the individual horse by using a belt speed to suit each individual. Ideally, the training programmes would have been standardised, but optimal training needs to be individualized for each horse, so a standardized programme would not have been appropriate and could have potentially caused problems for individual horses. All venues decreased the speed with increasing water depth [28] and adhered to WTl user guidelines [28]. Neither frequency of exposure to WT exercise nor the training programme outside of WT exercise were controlled, although all horses were engaged in regular work and remained engaged in work throughout the course of the study. It should be noted that the majority of the horses took part in dressage. The limitations of 2D videography and the occlusion of the upper limb for measurement of protraction–retraction must also be acknowledged.

## 5. Conclusions

Regular WT exercise within a sport horse training programme is associated with changes in technique for walking on a WT that reflect adaptation to movement in water, and this response may be influenced by the previous experience of the horses with WT exercise, the frequency of WT exercise sessions, and the type of WT exercise session used during training. This suggests that WT exercise sessions could be designed in accordance with specific training goals when used within a normal sport horse riding training programme.

## Figures and Tables

**Table 1:** Summary of the model for limb kinematics during the standardised water treadmill exercise test with peak carpal, metacarpophalangeal, tarsal, metatarsophalangeal flexion, peak forelimb protraction, retraction, and peak hindlimb protraction and retraction as outcome variables and test water depth and speed, week, prior treadmill experience, treadmill training in between tests (water depth, belt speed, and training frequency) as predictors. Horse was included as a random factor to account for clustering due to repeated measures. CB = coronary band, MCPJ = metacarpophalangeal joint, PMC = proximal metacarpal region, DR = distal radius, MTPJ = metatarsophalangeal joint, WT = water treadmill.

Outcome Measure		Regression Coefficient	Standard Error	95% Confidence Interval of Regression Coefficient	*p*-Value
Predictor Variable	Comparator for Interpretation				
**Peak flexion carpus**		**(°)**			
Intercept	*Baseline peak carpal flexion*	*131.64*	*9.8*		*-*
Week 20	Compared to week 0	−5.81	0.66	−7.11–4.51	<0.001
Week 40		−2.15	0.75	−3.63–0.68	0.004
Water depth—CB	Versus dry belt as baseline	−6.25	0.84	−7.90–4.59	<0.001
Water depth—MCPJ		−14.35	0.85	−16.02–12.68	<0.001
Water depth—PMC		−18.29	0.89	−20.03–16.54	<0.001
Water depth—DR		−19.82	0.94	−21.68–17.96	<0.001
Time using WT for 79–104 weeks	Versus less than 5 weeks as baseline	−7.89	3.67	−15.09–0.70	0.031
Test speed	Per metre per second of speed	3.28	1.6	0.14–6.43	0.04
**Peak flexion MCPJ**					
Intercept	*Baseline peak MCPJ flexion*	*170.74*	*14.82*		*-*
Week 20	Compared to week 0	−5.27	1.2	−7.63–2.91	<0.001
Water depth—CB	Versus dry belt as baseline	−9.97	1.46	−12.84–7.10	<0.001
Water depth—MCPJ		−15.18	1.49	−18.10–12.26	<0.001
Water depth—PMC		−19.68	1.63	−22.88–16.48	<0.001
Water depth—DR		−22.45	1.73	−25.88–19.02	<0.001
**Forelimb protraction**					
Intercept	*Baseline forelimb protraction*	*29.51*	3.49		*-*
Week 40	Compared to week 0	1.63	2.63	1.12–2.14	<0.001
Water depth—CB	Versus dry belt as baseline	1.3	0.28	0.74–1.87	<0.001
Training speed	Per metre per second of speed	−1.67	0.62	−2.89–0.44	0.008
**Forelimb retraction**					
Intercept	*Baseline forelimb retraction*	29.01	3.36		
Week 40	Compared to week 0	−1.72	0.22	−2.17–−1.79	<0.001
Water depth—CB	Versus dry belt as baseline	1.7	0.24	1.22–2.19	<0.001
Water depth—MCPJ		3.63	0.25	3.12–4.13	<0.001
Water depth—PMC		4.27	0.27	3.73–4.81	<0.001
Water depth—DR		4.51	0.29	3.94–5.09	<0.001
Once-a-week WT exercise	Versus baseline fortnightly	−1.33	0.61	−2.54–−0.13	0.029
Training speed	Per metre per second of speed	3.28	0.65	0.23–2.80	0.021
**Peak flexion tarsus**		**(°)**			
Intercept	*Baseline peak tarsal flexion*	*107.5*	*21.76*		*-*
Week 20	Compared to week 0	12.49	1.15	10.22–14.76	<0.001
Week 40		15.18	1.28	12.67–17.69	0.004
Water depth—CB	Versus dry belt as baseline	−13.98	1.45	−16.83–−11.14	<0.001
Water depth—MCPJ		−24.8	1.46	−27.67–−21.92	<0.001
Water depth—PMC		−29.39	1.52	−32.38–−26.41	<0.001
Water depth—DR		−30.09	1.63	−33.29–−26.88	<0.001
Three-times-a-month WT exercise	Versus baseline fortnightly	−8.89	4.38	−17.49–−0.30	0.042
Training at below carpus depth	Compared to MCPJ as baseline	14.21	5.82	2.79–25.63	0.015
**Peak flexion MTPJ**					
Intercept	*Baseline peak MTPJ flexion*	*156.46*	15.9		*-*
Week 20	Compared to week 0	−5.18	1.16	−7.47–−2.90	<0.001
Water depth—CB	Versus dry belt as baseline	−10.39	1.43	−13.20–−7.58	<0.001
Water depth—MCPJ		−18.38	1.45	−24.24–−15.53	<0.001
Water depth—PMC		−23.61	1.53	−26.67–−20.66	<0.001
Water depth—DR		−28.2	1.67	−31.48–−24.93	<0.001
Training at carpal depth	Compared to MCPJ as baseline	12.54	4.58	3.56–21.52	0.006
Using WT 5–24 weeks	Versus less than 5 weeks as baseline	12.41	6.27	−0.11–24.72	0.0048
Using WT 25–52 weeks		11.93	5.29	1.55–22.31	0.024
**Hindlimb protraction**					
Intercept	*Baseline hindlimb protraction*	*15.41*	4.29		*-*
Week 20	Compared to week 0	0.92	0.3	0.32–1.52	0.002
Week 40		1.33	0.34	0.65–2.01	<0.001
Water depth—CB	Versus dry belt as baseline	0.88	0.37	0.14–1.61	0.019
Water depth—MCPJ		2.15	0.39	1.38–2.91	<0.001
Water depth—PMC		1.58	0.4	0.79–2.37	<0.001
Training at carpal depth	Compared to MCPJ as baseline	2.92	1.39	0.18–5.66	0.037
**Hindlimb retraction**					
Intercept	*Baseline hindlimb retraction*	*38.82*	*3.95*		*-*
Week 20	Compared to week 0	−0.77	0.26	−1.29–−0.25	0.003
Week 40		−1.85	0.3	−2.44–−1.26	<0.001
Water depth—CB	Versus dry belt as baseline	1.84	0.32	1.22–2.19	<0.001
Water depth—MCPJ		3.07	0.34	3.12–4.13	<0.001
Water depth—PMC		3.49	0.35	3.73–4.81	<0.001
Water depth—DR		4.30	0.38	3.65–5.05	<0.001
Training at carpal depth	Compared to MCPJ as baseline	4.29	0.38	3.94–5.09	<0.001

**Table 2:** Summary of the model for dorsal midline and pelvic displacements during the standardised water treadmill exercise test with poll, wither, sacrum, and left and right tuber coxae range of motion as outcome variables and test water depth and speed, week, prior treadmill experience, treadmill training in between tests (water depth, belt speed, and training frequency), horses’ level, and discipline as predictors. Horse was included as a random factor to account for clustering due to repeated measures. CB = coronary band, MCPJ = metacarpophalangeal joint, PMC = proximal metacarpal region, DR = distal radius, WT = water treadmill, ROM = range of motion, LTC = left tuber coxae, RTC = right tuber coxae; mm = millimetre.

*Outcome Measure*		Regression Coefficient	Standard Error	95% Confidence Interval of Regression Coefficient	*p*-Value
Predictor Variable	Comparator for Interpretation				
***Poll***		**(mm)**			
**Dorsoventral**	*Baseline dorsoventral ROM*	*99.38*	*1.7*		*-*
*Intercept*					
Water depth—CB	Versus dry belt as baseline	10.71	2.62	5.57–15.86	<0.001
Water depth—MCPJ		18.73	2.61	13.80–24.05	<0.001
Water depth—PMC		33.77	2.63	28.60–38.95	<0.001
Water depth—DR		43.22	2.63	38.05–48.40	<0.001
**Mediolateral**	*Baseline mediolateral ROM*	*59.3*	*0.92*		*-*
*Intercept*					
Week 40	Compared to week 0	9.69	1.61	6.53–12.84	<0.001
Water depth—CB	Versus dry belt as baseline	8.3	1.86	4.65–11.95	0.002
Water depth—MCPJ		14.02	1.85	10.38–17.66	0.018
Water depth—PMC		23.08	1.87	19.41–26.75	0.001
Water depth—DR		25.55	1.87	21.87–29.21	0.045
**Craniocaudal**	*Baseline craniocaudal ROM*	*118.28*	*1.71*		*-*
*Intercept*					
Week 20	Compared to week 0	−7.14	2.04	−11.16–−3.13	<0.001
Week 40	Compared to week 0	−9.4	2.3	−13.91–−4.89	<0.001
Water depth—CB	Versus dry belt as baseline	23.85	2.64	18.66–29.05	<0.001
Water depth—MCPJ		40.95	2.64	35.77–46.13	<0.001
Water depth—PMC		63.13	2.66	57.91–68.35	<0.001
Water depth—DR		73.54	2.66	68.31–78.76	<0.001
Training speed	Per metre per second of speed	16.58	7.22	2.54–30.72	0.021
***Withers***		**(mm)**			*-*
**Dorsoventral**	*Baseline dorsoventral ROM*	*60.93*	*0.83*		
*Intercept*					
Water depth—CB	Versus dry belt as baseline	14.2	1.05	12.14–16.26	<0.001
Water depth—MCPJ		23	1.04	20.94–25.05	<0.001
Water depth—PMC		28.43	1.05	26.35–30.50	<0.001
Water depth—DR		31.28	1.05	29.20–33.36	<0.001
Weekly WT exercise	Versus baseline fortnightly	−9.62	3.7	−16.88–−2.35	0.009
**Mediolateral**	*Baseline mediolateral ROM*	*49.12*	*0.73*		*-*
*Intercept*					
Week 20	Compared to week 0	3.3	1.25	0.85–5.75	0.008
Week 40	Compared to week 0	4.12	1.33	1.50–6.74	0.002
Water depth—MCPJ	Versus dry belt as baseline	7.02	1.56	3.95–10.09	<0.001
Water depth—PMC		14.88	1.58	11.78–17.78	<0.001
Water depth—DR		18.81	1.58	15.70–21.91	<0.001
Three-times-per-month WT exercise	Versus baseline fortnightly	−20.17	8.63	−37.09–−3.26	0.019
**Craniocaudal**	*Baseline craniocaudal ROM*	*46.28*	*0.48*		*-*
*Intercept*					
Week 40	Compared to week 0	−2.78	0.75	−4.26–−1.29	<0.001
Water depth—CB	Versus dry belt as baseline	6.92	0.88	5.19–8.65	<0.001
Water depth—MCPJ		9.94	0.87	8.23–11.66	<0.001
Water depth—PMC		9.34	0.88	7.60–11.08	<0.001
Water depth—DR		8.4	0.88	6.65–10.00	<0.001
Weekly WT exercise	Versus baseline fortnightly	−5.8	2.66	−11.01–−0.58	0.029
***Sacrum***		**(mm)**			
**Dorsoventral**	*Baseline dorsoventral ROM*	*89.53*	*0.95*		*-*
*Intercept*					
Week 20	Compared to week 0	−3.75	0.9	−5.53–−1.97	<0.001
Water depth—CB	Versus dry belt as baseline	17.56	1.17	15.27–19.86	<0.001
Water depth—MCPJ		28.39	1.16	26.10–30.68	<0.001
Water depth—PMC		35.91	1.17	33.60–38.22	<0.001
Water depth—DR		39.93	1.17	37.62–38.22	<0.001
Three-times-per-month WT exercise	Versus baseline fortnightly	−10.16	3.8	−17.61–−2.70	0.008
Using WT for 5–24 weeks	Versus less than 5 weeks as baseline	−20.51	9.99	−40.09–−0.92	0.04
*Intercept*	*Baseline mediolateral ROM*	*66.84*	*0.89*		*-*
Water depth—CB	Versus dry belt as baseline	10.92	1.29	8.38–13.47	<0.001
Water depth—MCPJ	Compared to week 0	19.97	1.29	17.43–22.51	<0.001
Water depth—PMC	Versus dry belt as baseline	29.75	1.3	27.18–32.30	<0.001
Water depth—DR		35.49	1.3	32.93–38.05	<0.001
**Craniocaudal**	*Baseline craniocaudal ROM*	*47.86*	*0.58*		*-*
*Intercept*					
Water depth—CB	Versus dry belt as baseline	4.84	0.99	2.89–6.78	<0.001
Water depth—MCPJ		5.78	0.99	3.84–7.73	<0.001
Water depth—PMC		3.7	1	1.74–5.66	<0.001
Water depth—DR		2.02	0.99	0.06–3.98	0.043
***LTC***		**(mm)**			
**Dorsoventral**	*Baseline dorsoventral ROM*	*108.45*	*10.03*		*-*
*Intercept*					
Week 20	Compared to week 0	−3.03	1.19	−5.58–−0.68	0.011
Water depth—CB	Versus dry belt as baseline	15.33	1.43	12.52–18.14	<0.001
Water depth—MCPJ		26.85	1.43	24.04–29.65	<0.001
Water depth—PMC		34.26	1.44	31.42–37.10	<0.001
Water depth—DR		37.68	2.44	34.84–40.51	<0.001
Test speed	Versus baseline fortnightly	6.06	2.67	0.83–11.30	0.023
Using WT for 5–24 weeks	Versus less than 5 weeks as baseline	−22.72	11.53	−45.33–−0.10	0.049
**Mediolateral**	*Baseline mediolateral ROM*	*52.93*	*0.72*		*-*
*Intercept*					
Water depth—CB	Versus dry belt as baseline	10.43	1.13	8.21–12.64	<0.001
Water depth—MCPJ		16.21	1.12	14.00–18.42	<0.001
Water depth—PMC		22.21	1.14	19.98–24.45	<0.001
Water depth—DR		25.16	1.13	22.93–27.40	<0.001
**Craniocaudal**	*Baseline craniocaudal ROM*	*57.6*	*0.59*		*-*
*Intercept*					
Week 40	Compared to week 0	−1.75	0.86	−3.34–−0.05	0.043
Water depth—CB	Versus dry belt as baseline	2.49	1.03	0.47–4.51	0.016
Water depth—MCPJ		3.22	1.02	1.21–5.42	0.002
***RTC***		**(mm)**			
**Dorsoventral**	*Baseline dorsoventral ROM*	*110.14*	*1*		*-*
*Intercept*					
Water depth—CB	Versus dry belt as baseline	17.05	1.15	14.78–19.81	<0.001
Water depth—MCPJ		28.32	1.15	26.06–30.58	<0.001
Water depth—PMC		35.5	1.16	33.21–37.78	<0.001
Water depth—DR		38.11	1.16	35.83–40.39	<0.001
**Mediolateral**	*Baseline mediolateral ROM*	*56.53*	*0.8*		*-*
*Intercept*					
Week 20	Compared to week 0	5.31	1.03	3.28–7.35	<0.001
Week 40	Compared to week 0	2.96	1.11	0.78–5.14	0.008
Water depth—CB	Versus dry belt as baseline	11.45	1.13	8.21–12.64	<0.001
Water depth—MCPJ	Compared to week 0	18.14	1.12	14.00–18.42	<0.001
Water depth—PMC	Versus dry belt as baseline	24.86	1.14	19.98–24.45	<0.001
Water depth—DR		28.34	1.13	22.93–27.40	<0.001
Once-a-week WT exercise	Versus baseline fortnightly	−7.17	3.58	−14.21–−0.14	0.045
**Craniocaudal**	*Baseline craniocaudal ROM*	59.45	*0.54*		*-*
*Intercept*					
Week 40	Compared to week 0	−3.91	0.92	−5.73–−2.10	<0.001
Water depth—CB	Versus dry belt as baseline	3.66	1.08	1.54–5.79	0.001
Water depth—MCPJ		4.23	1.08	2.11–6.35	<0.001
Water depth—PMC		3.58	1.09	1.44–5.72	0.001

**Table 3:** Summary of the model for flexion–extension and lateral bending range of motion during the water treadmill exercise test, with flexion–extension and lateral bending of the 10th, 13th, and 18th thoracic vertebrae (T10, T13, and T18, respectively) and the 3rd and 5th lumbar vertebrae (L3 and L5, respectively) as outcome variables and test water depth and speed, week, prior treadmill experience, treadmill training in between tests (water depth, belt speed, and training frequency), horses’ level, and discipline as predictors. Horse was included as a random factor to account for clustering due to repeated measures. CB = coronary band, MCPJ = metacarpophalangeal joint, PMC = proximal metacarpal region, DR = distal radius, midcannon = mid-proximal to distal metacarpus/metatarsus, WT = water treadmill, ROM = range of motion.

*Outcome Measure*	Comparator for Interpretation	Regression Coefficient	Standard Error	95% Confidence Interval of Regression Coefficient	*p*-Value
Predictor Variable					
***T10 ROM***		**(°)**			
***Flexion–extension***					
*Intercept*	*Baseline flexion–extension ROM*	*8.81*	*0.1*		*-*
Water depth—CB	Versus dry belt as baseline	4.02	0.19	3.64–4.41	<0.001
Water depth—MCPJ		1.95	0.19	1.56–2.34	<0.001
Water depth—PMC		4.46	0.2	4.06–4.87	<0.001
Water depth—DR		3.26	0.21	2.83–3.69	<0.001
Using WT for 5–24 weeks	Versus less than 5 weeks as baseline	−2.59	0.96	−4.48–0.71	0.007
***Lateral bending***					
*Intercept*	*Baseline lateral bending ROM*	*10.84*	*0.1*		*-*
Water depth—CB	Versus dry belt as baseline	−0.76	0.24	−1.22–0.28	0.002
Water depth—MCPJ		0.27	0.24	0.98–1.04	0.018
Water depth—DR		0.83	0.26	2.83–3.69	0.001
Weekly WT exercise	Versus baseline fortnightly	−0.94	0.47	−1.86–−0.20	0.045
***T13 ROM***		**(°)**			
***Flexion–extension***					
*Intercept*	*Baseline flexion–extension ROM*	*8.16*	*0.11*		*-*
Water depth—CB	Versus dry belt as baseline	−1.32	0.16	−1.65–−1.00	<0.001
Water depth—MCPJ		2.25	0.16	1.93–2.58	<0.001
Water depth—PMC		−1	0.17	−1.35–−0.66	<0.001
Water depth—DR		3.64	0.18	3.29–4.01	<0.001
***Lateral bending***		**(°)**			
*Intercept*	*Baseline lateral bending ROM*	*9.23*	*0.14*		*-*
Test speed	Per metre per second of speed	0.95	0.4	0.16–1.74	0.018
Water depth—CB	Versus dry belt as baseline	4.99	0.19	4.60–5.37	<0.001
Water depth—MCPJ		0.4	0.19	0.13–0.80	0.043
Water depth—PMC		5.57	0.2	5.16–5.97	<0.001
Water depth—DR		0.87	0.21	0.45–1.30	<0.001
***T18 ROM***		**(°)**			
***Flexion–extension***					
*Intercept*	*Baseline flexion–extension ROM*	*5.11*	*0.12*		*-*
Week 20	Compared to week 0	1.75	0.24	1.26–2.23	<0.001
Week 40	Compared to week 0	0.91	0.25	0.40–1.42	<0.001
Water depth—CB	Versus dry belt as baseline	−0.69	0.31	−1.30–−0.87	<0.001
Water depth—MCPJ		0.71	0.31	0.95–1.32	<0.001
Water depth—DR		1.66	0.33	1.00–2.32	<0.001
***Lateral bending***		** (°) **			
*Intercept*	*Baseline lateral bending ROM*	*5.78*	*0.12*		*-*
Week 20	Compared to week 0	1.83	0.25	1.33–2.33	<0.001
Week 40	Compared to week 0	0.69	0.26	0.17–1.20	0.009
Water depth—CB	Versus dry belt as baseline	1.65	0.31	1.03–2.27	<0.001
Water depth—PMC		1.93	0.32	1.29–2.57	<0.001
Training depth—midcannon	Compared to MCPJ as baseline	1.81	0.89	0.60–3.57	0.043
Using WT for 5–24 weeks	Versus less than 5 weeks as baseline	−1.74	0.86	−3.42–−0.52	0.043
Using WT for 52–78 weeks	Versus less than 5 weeks as baseline	−2.11	0.87	−3.83–−0.40	0.015
***L3 ROM***		**(°)**			
***Flexion–extension***					
*Intercept*	*Baseline flexion–extension ROM*	*7.61*	*0.11*		*-*
Test speed	Per metre per second of speed	1.31	0.37	0.39–1.86	0.002
Water depth—CB	Versus dry belt as baseline	−1.33	0.2	−1.72–−0.93	<0.001
Water depth—MCPJ		1.85	0.2	1.45–2.25	<0.001
Water depth—PMC		0.79	0.21	−1.20–−0.37	<0.001
Water depth—DR		3.01	0.22	2.57–3.45	<0.001
***Lateral bending***		** (°) **			
*Intercept*	*Baseline lateral bending ROM*	*8.05*	*0.12*		*-*
Water depth—CB	Versus dry belt as baseline	4.17	0.17	3.82–4.51	<0.001
Water depth—PMC		4.41	0.18	4.04–4.47	<0.001
***L5 ROM***		**(°)**			
***Flexion–extension***					
*Intercept*	*Baseline flexion–extension ROM*	*7.73*	*0.09*		*-*
Water depth—CB	Versus dry belt as baseline	1.17	0.21	0.75–−1.58	<0.001
Water depth—MCPJ		1.32	0.21	0.91–1.74	<0.001
Water depth—PMC		2.08	0.22	1.64–2.51	<0.001
Water depth—DR		1.87	0.23	1.42–2.33	<0.001
***Lateral bending***		**(°)**			
*Intercept*	*Baseline lateral bending ROM*	*8.72*	*0.09*		*-*
Water depth—CB	Versus dry belt as baseline	−0.74	0.18	−1.11–−0.37	<0.001
Water depth—MCPJ		0.45	0.18	0.09–0.82	0.015
Water depth—PMC		−0.85	0.19	−1.22–−0.46	<0.001
Water depth—DR		0.58	0.2	0.18–0.99	0.004
Training depth—midcannon	Compared to MCPJ as baseline	1.53	0.75	0.54–3.01	0.042

## Data Availability

Data are unavailable due to privacy or ethical restrictions.

## Supplementary Materials

The following supporting information can be downloaded at: https://www.mdpi.com/article/10.3390/ani14162393/s1, Table S1: Summary of non-significant predictors of limb kinematics during the standardised water treadmill exercise test, with peak carpal, metacarpophalangeal, tarsal, and metatarsophalangeal flexion; peak forelimb protraction and retraction; and peak hindlimb protraction and retraction as outcome variables and test water depth and speed, week, prior treadmill experience, and treadmill training in between tests (water depth, belt speed, and training frequency) as predictors. Table S2: Summary of non-significant predictors of upper body displacements during the standardised water treadmill exercise test, with poll, wither, sacrum, and left and right tuber coxae (LTC/RTC) range of motion as outcome variables and test water depth and speed, week, prior treadmill experience, treadmill training in between tests (water depth, belt speed, and training frequency), horses’ level, and discipline as predictors. Table S3: Summary of non-significant predictors of flexion–extension and lateral bending range of motion during water treadmill exercise test, with the 10th, 13th, and 18th thoracic vertebrae (T10, T13, and T18, respectively) and the 3rd and 5th lumbar vertebrae (L3 and L5, respectively) flexion–extension and lateral bending as outcome variables and test water depth and speed, week, prior treadmill experience, treadmill training in between tests (water depth, belt speed, and training frequency), horses’ level, and discipline as predictors.
