# Supplementary material for: Kinematic Responses to Water Treadmill Exercise When Used Regularly within a Sport Horse Training Programme: A Longitudinal, Observational Study
Source: Animals (Basel). 2024 Aug 18;14(16):2393. doi: 10.3390/ani14162393 (PMC11350662; doi:10.3390/ani14162393)
Supplement: Supplementary file 1 [file animals-14-02393-s001.zip › animals-3140350-supplementary.pdf]

Table S1. Summary of non-significant predictors for limb kinematics during the standardised water treadmill exercise test with peak carpal, metacarpophalangeal, tarsal, metatarsophalangeal flexion, peak forelimb protraction, retraction, peak hindlimb protraction and retraction, as outcome variables and test water depth and speed, week, prior treadmill experience, treadmill training in between tests (water depth, belt speed and training frequency) as predictors. Horse was included as a random factor to account for clustering due to repeated measures. CB = coronary band, MCPJ = metacarpophalangeal joint, PMC = proximal metacarpal region, DR = distal radius, MTPJ = metatarsophalangeal joint, midcannon = mid-proximal to distal metacarpus/metatarsus, WT = water treadmill

Outcome measure **Predictor variable**	**Comparator for interpretation**	**Regression coefficient**	**Standard error**	**95% confidence interval of regression coefficient**	**P-value**
Peak flexion carpus		*(°)*			
Intercept	*Baseline peak carpal flexion*	*131.64*	*9.8*		-
Using WT 5- 24 weeks	Versus less than 5 weeks as baseline	-3.6	*4.3*	-12.02 – 4.82	*0.402*
Using WT for 25-52 weeks	Versus less than 5 weeks as baseline	-5.19	3.63	-12.30– 1.92	0.153
Using WT for 53-78 weeks	Versus less than 5 weeks as baseline	-6.44	4.32	-14.91– 2.02	0.136
Using WT for more than 104 weeks	Versus less than 5 weeks as baseline	-0.357	4.36	-12.12 – 4.97	0.412
WT exercise: Three times a month	Versus baseline fortnightly	2.23	2.89	-3.42 – 7.89	0.439
WT exercise: Once a month	Versus baseline fortnightly	-0.53	2.62	-5.67 –4.60	0.839
WT exercise: Weekly	Versus baseline fortnightly	1.68	1.65	-1.64–5.00	0.322
Training depth- midcannon	Compared to MCPJ as baseline	-0.86	2.77	-6.29– 4.56	0.754
Training depth- Below carpus	Compared to MCPJ as baseline	0.69	3.74	-6.64– 8.02	0.854
Training depth- Carpus	Compared to MCPJ as baseline	0.06	3.08	-5.98–6.09	0.985
Training speed	Per metre per second of speed	-1.47	2	-5.38– 2.44	0.461
Peak flexion MCPJ					
Intercept	*Baseline peak MCPJ flexion*	*170.74*	*14.82*		-
Using WT 5- 24 weeks	Versus less than 5 weeks as baseline	-4	*6.34*	-16.44 – 8.43	*0.528*
Using WT for 25-52 weeks	Versus less than 5 weeks as baseline	-3.82	5.36	-14.35– 6.69	0.476
Using WT for 53-78 weeks	Versus less than 5 weeks as baseline	-0.47	6.38	-12.98 – 12.03	0.941
Using WT for 79-104 weeks	Versus less than 5 weeks as baseline	-1.07	5.43	-11.71– 9.57	0.844
Using WT for more than 104 weeks	Versus less than 5 weeks as baseline	-11.87	6.42	-24.46 – 0.72	0.065
Week 40	Compared to week 0	-0.54	1.36	-3.20 –2.13	0.694
WT exercise: Once a month	Versus baseline fortnightly	6.38	3.86	-1.21 –13.96	0.099
Training depth- midcannon	Compared to MCPJ as baseline	0.85	4.11	-7.21– 8.91	0.59
Training depth- Below carpus	Compared to MCPJ as baseline	-0.73	5.6	-11.70– 10.23	0.559
Training depth- Carpus	Compared to MCPJ as baseline	-4.91	4.6	-13.93– 4.10	0.579
Test speed	Per metre per second of speed	3.44	1.93	-2.39– 7.80	0.299
Training speed	Per metre per second of speed	-4.15	4.17	-6.19– 5.51	0.91
Forelimb protraction					
Intercept	*Baseline forelimb protraction*	*29.51*	3.49		-
Using WT 5- 24 weeks	Versus less than 5 weeks as baseline	-2.5	*1.57*	-5.57 – 0.56	*0.11*

Using WT for 25-52 weeks	Versus less than 5 weeks as baseline	-1.78	1.32	-4.37– 0.81	0.177
Using WT for 53-78 weeks	Versus less than 5 weeks as baseline	-0.99	1.57	-4.08 – 2.09	0.527
Using WT for 79-104 weeks	Versus less than 5 weeks as baseline	-1.29	1.34	-3.91– 1.32	0.333
Using WT for more than 104 weeks	Versus less than 5 weeks as baseline	-1.51	1.6	-4.64 – 1.62	0.345
Week 20	Compared to week 0	-0.07	0.24	-0.53 –0.39	0.765
Water depth- PMC	Versus dry belt as baseline	0.97	0.31	-0.51 – 0.71	0.754
Water depth- DR	Versus dry belt as baseline	-0.3	0.33	-0.96– 0.34	0.36
WT exercise: Three times a month	Versus baseline fortnightly	0.81	1	-1.16 – 2.77	0.774
WT exercise: Once a month	Versus baseline fortnightly	0.09	1.36	-2.58 –2.77	0.599
WT exercise: Weekly	Versus baseline fortnightly	1.24	1.12	-0.96–3.44	0.826
Training depth- midcannon	Compared to MCPJ as baseline	-3.12	5.8	-14.50– 8.25	0.42
Training depth- Below carpus	Compared to MCPJ as baseline	4.56	7.79	-10.71– 19.83	0.945
Training depth- Carpus	Compared to MCPJ as baseline	-3.56	6.41	-16.14– 9.01	0.268
Test speed	Per metre per second of speed	0.18	0.55	-0.91– 1.26	0.75
Training speed	Per metre per second of speed	-1.32	0.73	-2.76– 0.10	0.069
Forelimb retraction					
Intercept	*Baseline forelimb retraction*	29.01	3.36		
Using WT 5- 24 weeks	Versus less than 5 weeks as baseline	0.63	*1.61*	-2.52 – 3.79	*0.696*
Using WT for 25-52 weeks	Versus less than 5 weeks as baseline	0.64	1.36	-2.01– 3.31	0.635
Using WT for 53-78 weeks	Versus less than 5 weeks as baseline	-0.65	1.61	-3.80 – 2.51	0.688
Using WT for 79-104 weeks	Versus less than 5 weeks as baseline	1.14	1.37	-1.55– 3.83	0.405
Using WT for more than 104 weeks	Versus less than 5 weeks as baseline	0.06	1.69	-3.37 – 3.24	0.970
Week 20	Compared to week 0	0.01	0.2	-0.39 –0.41	0.95
WT exercise: Three times a month	Versus baseline fortnightly	-0.07	1.12	-2.26 –2.12	0.679
WT exercise: Once a month	Versus baseline fortnightly	0.98	0.99	-2.93 –0.97	0.323
Training depth- midcannon	Compared to MCPJ as baseline	0.41	1.03	-1.60– 2.44	0.685
Training depth- Below carpus	Compared to MCPJ as baseline	-0.5	1.41	-3.27– 2.27	0.722
Training depth- Carpus	Compared to MCPJ as baseline	0.08	1.15	-2.17– 2.34	0.942
Test speed	Per metre per second of speed	0.03	0.52	-0.99– 1.05	0.075
Peak flexion tarsus		*(°)*			
Intercept	*Baseline peak tarsal flexion*	*107.5*	*21.76*		-
Using WT 5- 24 weeks	Versus less than 5 weeks as baseline	1.79	*6.34*	-10.82 – 14.40	*0.781*
Using WT for 25-52 weeks	Versus less than 5 weeks as baseline	-2.03	5.42	-12.67– 8.58	0.707
Using WT for 53-78 weeks	Versus less than 5 weeks as baseline	-1.83	6.49	-14.55 – 10.87	0.777
Using WT for 79-104 weeks	Versus less than 5 weeks as baseline	-6.42	5.5	-17.20– 4.34	0.242
Using WT for more than 104 weeks	Versus less than 5 weeks as baseline	2.26	6.54	-10.55 – 15.07	0.279
WT exercise: Weekly	Versus baseline fortnightly	0.37	2.55	-5.36 –4.62	4.62
WT exercise: Once a month	Versus baseline fortnightly	2.87	3.93	-10.58 –4.83	4.83
Training depth- midcannon	Compared to MCPJ as baseline	-3.68	4.17	-11.86– 4.49	0.377
Training depth- Carpus	Compared to MCPJ as baseline	1.55	4.71	-7.67– 10.77	0.742
Test speed	Per metre per second of speed	3.38	2.61	-1.73– 8.49	0.169
Training speed	Per metre per second of speed	-1.91	3.04	-7.87– 4.04	0.528

Peak flexion MTPJ					
Intercept	*Baseline peak MTPJ flexion*	*156.46*	15.9		-
Using WT for 53-78 weeks	Versus less than 5 weeks as baseline	8.8	6.32	-3.58 – 21.19	0.164
Using WT for 79-104 weeks	Versus less than 5 weeks as baseline	8.23	5.37	-2.29– 18.75	0.125
Using WT for more than 104 weeks	Versus less than 5 weeks as baseline	3.48	6.37	-9.00– 15.97	0.585
Week 40	Compared to week 0	-0.2	1.29	-2.74 –2.33	0.875
Water depth- PMC	Versus dry belt as baseline	1.25	1.04	-0.79 – 3.28	0.23
Water depth- DR	Versus dry belt as baseline	-0.78	1.04	-2.81– 1.25	0.451
WT exercise: Three times a month	Versus baseline fortnightly	-0.92	4.27	-9.30 – 7.46	0.83
WT exercise: Once a month	Versus baseline fortnightly	3.44	3.83	-4.05 –10.94	0.367
WT exercise: Weekly	Versus baseline fortnightly	3.95	2.49	-0.94–8.82	0.113
Training depth- midcannon	Compared to MCPJ as baseline	5.44	4.08	-2.55– 13.45	0.59
Training depth- PMC	Compared to MCPJ as baseline	9.17	5.55	-1.70– 20.05	0.182
Test speed	Per metre per second of speed	2.87	2.59	-2.20– 7.94	0.268
Training speed	Per metre per second of speed	-4.75	2.95	-10.54– 1.03	0.107
Hindlimb protraction					
Intercept	*Baseline hindlimb protraction*	*15.41*	4.29		-
Using WT 5- 24 weeks	Versus less than 5 weeks as baseline	-1.03	*9.01*	-4.92 – 2.84	*0.601*
Using WT for 25-52 weeks	Versus less than 5 weeks as baseline	-0.45	7.62	-3.74– 2.83	0.788
Using WT for 53-78 weeks	Versus less than 5 weeks as baseline	0.27	9.02	-3.62 – 4.17	0.891
Using WT for 79-104 weeks	Versus less than 5 weeks as baseline	1.04	7.6	-2.27– 4.36	0.538
Using WT for more than 104 weeks	Versus less than 5 weeks as baseline	2.34	9.2	-1.59 – 6.28	0.243
Water depth- DR	Versus dry belt as baseline	0.67	0.43	-0.18– 1.51	0.124
WT exercise: Three times a month	Versus baseline fortnightly	-0.89	1.32	-3.47 – 1.70	0.5
WT exercise: Once a month	Versus baseline fortnightly	0.01	1.2	-2.35 –2.34	0.997
WT exercise: Weekly	Versus baseline fortnightly	-0.94	0.77	-2.46–0.57	0.224
Training depth- midcannon	Compared to MCPJ as baseline	0.61	1.28	-1.89– 3.11	0.635
Training depth- PMC	Compared to MCPJ as baseline	3.08	1.72	-0.28– 6.44	0.073
Test speed	Per metre per second of speed	1.05	0.72	-0.35– 2.45	0.144
Training speed	Per metre per second of speed	-0.08	0.91	-1.87– 1.71	0.93
Hindlimb retraction					
Intercept	*Baseline hindlimb retraction*	*38.82*	*3.95*		-
Using WT 5- 24 weeks	Versus less than 5 weeks as baseline	-0.68	*1.99*	-4.57 – 3.21	*0.732*
Using WT for 25-52 weeks	Versus less than 5 weeks as baseline	0.71	1.68	-2.58– 3.99	0.674
Using WT for 53-78 weeks	Versus less than 5 weeks as baseline	-0.92	1.99	-4.83 – 2.99	0.643
Using WT for 79-104 weeks	Versus less than 5 weeks as baseline	0.51	1.7	-2.81– 3.83	0.762
Using WT for more than 104 weeks	Versus less than 5 weeks as baseline	-1.05	2.02	-5.01 – 2.91	0.601
WT exercise: Three times a month	Versus baseline fortnightly	1.88	1.35	-0.77– 4.51	0.165
WT exercise: Once a month	Versus baseline fortnightly	0.25	1.22	-2.63 –2.14	0.84
WT exercise: Weekly	Versus baseline fortnightly	0.73	0.78	-2.25–0.80	0.352
Training depth- midcannon	Compared to MCPJ as baseline	-0.05	1.28	-2.55– 2.45	0.97

Training depth- Below carpus	Compared to MCPJ as baseline	-0.85	1.74	-5.25– 1.55	0.287
Test speed	Per metre per second of speed	1.26	0.66	-0.02– 2.55	0.054
Training speed	Per metre per second of speed	1.03	0.93	-0.78– 2.84	0.264

Table S2. Summary of non-significant predictors for upper body displacements during the standardised water treadmill exercise test with poll, wither, sacrum, left and right tuber coxae (LTC/RTC) range of motion as outcome variables and test water depth and speed, week, prior treadmill experience, treadmill training in between tests (water depth, belt speed and training frequency), horses' level and discipline as predictors. Horse was included as a random factor to account for clustering due to repeated measures. CB = coronary band, MCPJ = metacarpophalangeal joint, PMC = proximal metacarpal region, DR = distal radius, midcannon = mid-proximal to distal metacarpus/metatarsus, WT = water treadmill, ROM = range of motion, mm = millimetre.

Outcome measure					
**Predictor variable**	**Comparator for interpretation**	**Regression coefficient**	**Standard error**	**95% confidence interval of regression coefficient**	**P-value**
Poll		***(mm)***			
Intercept	***Baseline dorsoventral ROM***	*99.38*	*1.7*		-
Using WT 5- 24 weeks	Versus less than 5 weeks as baseline	-13.66	27.33	-67.23 – 39.90	0.617
Using WT for 25-52 weeks	Versus less than 5 weeks as baseline	-6.72	23.12	-52.03 – 38.60	0.771
Using WT for 53-78 weeks	Versus less than 5 weeks as baseline	-8.45	27.34	-62.04 – 45.13	0.757
Using WT for 79-104 weeks	Versus less than 5 weeks as baseline	-7.04	23.35	-52.81 – 38.71	0.763
Using WT for more than 104 weeks	Versus less than 5 weeks as baseline	9.18	27.94	-45.57 – 63.94	0.742
Week 20	Compared to week 0	0.85	2.03	-3.13 – 4.83	0.676
Week 40	Compared to week 0	-1.98	2.28	-6.46 – 2.50	0.387
WT exercise: Three times a month	Versus baseline fortnightly	-6.01	10.65	-26.90 – 14.84	0.572
WT exercise: Once a month	Versus baseline fortnightly	6.98	16.68	-25.71 – 36.69	0.675
WT exercise: Weekly	Versus baseline fortnightly	-17.73	18.01	-53.03 – 17.56	0.325
Training depth- midcannon	Compared to MCPJ as baseline	15.86	17.47	-18.37– 50.11	0.364
Training depth- Below carpus	Compared to MCPJ as baseline	30.19	23.56	-15.98– 176.37	0.2
Training depth- Carpus	Compared to MCPJ as baseline	15.68	19.36	-22.26– 53.62	0.418
Test speed	Per metre per second of speed	-7.15	5.13	-17.20 – 2.91	0.164
Training speed	Per metre per second of speed	17.03	12.62	-7.70 – 41.75	0.177
Intercept	***Baseline mediolateral ROM***	*59.3*	*0.92*		-
Using WT 5- 24 weeks	Versus less than 5 weeks as baseline	-9.65	*10.16*	-29.57 – 10.27	*0.343*
Using WT for 25-52 weeks	Versus less than 5 weeks as baseline	-6.98	8.59	-23.82 – 9.86	0.417
Using WT for 53-78 weeks	Versus less than 5 weeks as baseline	2.64	10.22	-17.38 – 22.68	0.769
Using WT for 79-104 weeks	Versus less than 5 weeks as baseline	1.3	8.7	-15.73 – 18.35	0.881
Using WT for more than 104 weeks	Versus less than 5 weeks as baseline	5.46	5.46	-14.85 – 25.77	0.598
Week 20	Compared to week 0	2	1.43	-0.81 – 4.80	0.163
WT exercise: Three times a month	Versus baseline fortnightly	-4.34	3.98	-12.15 – 3.45	0.275

WT exercise: Once a month	Versus baseline fortnightly	-3.32	6.24	-15.51 – 8.90	0.594
WT exercise: Weekly	Versus baseline fortnightly	10.06	6.79	-3.24 –23.36	0.138
Training depth- midcannon	Compared to MCPJ as baseline	5.97	6.53	-6.81– 18.76	0.36
Training depth- Below carpus	Compared to MCPJ as baseline	-3.57	8.8	-20.83– 13.67	0.685
Training depth- Carpus	Compared to MCPJ as baseline	2	7.29	-21.28– 16.28	0.784
Test speed	Per metre per second of speed	-2.74	3.25	-9.09– 3.63	0.399
Training speed	Per metre per second of speed	7.55	4.73	-1.70 – 16.82	0.11
Intercept	***Baseline craniocaudal ROM***	*118.28*	*1.71*		-
Using WT 5- 24 weeks	Versus less than 5 weeks as baseline	15.62	*17.86*	-19.38 – 50.64	*0.382*
Using WT for 25-52 weeks	Versus less than 5 weeks as baseline	5.27	15.1	-24.33 – 34.87	0.727
Using WT for 53-78 weeks	Versus less than 5 weeks as baseline	19.96	17.91	-15.51 – 55.08	0.265
Using WT for 79-104 weeks	Versus less than 5 weeks as baseline	19.48	15.27	-10.44 – 49.41	0.202
Using WT for more than 104 weeks	Versus less than 5 weeks as baseline	2.21	18.23	-33.52 – 37.95	0.903
WT exercise: Three times a month	Versus baseline fortnightly	2.54	6.98	-16.23 – 11.14	0.716
WT exercise: Once a month	Versus baseline fortnightly	-11.42	10.94	-32.86 – 10.01	0.296
WT exercise: Weekly	Versus baseline fortnightly	-18.55	11.86	-41.8 –4.68	0.118
Training depth- midcannon	Compared to MCPJ as baseline	13.51	11.44	-8.92– 35.94	0.238
Training depth- Below carpus	Compared to MCPJ as baseline	22.65	15.44	-7.599– 52.93	0.142
Training depth- Carpus	Compared to MCPJ as baseline	9.91	12.73	-15.04– 34.86	0.436
Test speed	Per metre per second of speed	-2.43	4.86	-11.97– 7.10	0.616
Withers		***(mm)***			
Intercept	***Baseline dorsoventral ROM***	*60.93*	*0.83*		-
Using WT 5- 24 weeks	Versus less than 5 weeks as baseline	-11.78	*9.75*	-30.88 – 7.2	0.227
Using WT for 25-52 weeks	Versus less than 5 weeks as baseline	-2.48	8.25	-18.65 – 13.69	0.764
Using WT for 53-78 weeks	Versus less than 5 weeks as baseline	-7.61	9.75	-26.72 – 11.50	0.435
Using WT for 79-104 weeks	Versus less than 5 weeks as baseline	3.64	8.32	-12.66 – 19.97	0.661
Using WT for more than 104 weeks	Versus less than 5 weeks as baseline	2.87	9.96	-16.65 – 22.39	0.773
Week 20	Compared to week 0	0.7	0.83	-0.95 –2.35	0.676
Week 40	Compared to week 0	0.13	1.44	-0.47 – 3	0.405
WT exercise: Once a month	Versus baseline fortnightly	-7.4	5.94	-19.05 – 4.24	0.213
WT exercise: Weekly	Versus baseline fortnightly	-0.57	6.42	-12.64 – 12.52	0.993
Training depth- midcannon	Compared to MCPJ as baseline	9.4	6.27	-2.89– 21.70	0.134
Training depth- Below carpus	Compared to MCPJ as baseline	-0.18	8.43	-16.69– 16.34	0.983
Training depth- Carpus	Compared to MCPJ as baseline	4.95	6.94	-8.65– 18.55	0.476
Test speed	Per metre per second of speed	3.1	2.02	-0.85– 7.06	0.124
Training speed	Per metre per second of speed	3.35	4.5	-5.46 – 12.17	0.456
Intercept	***Baseline mediolateral ROM***	*49.12*	*0.73*		-
Using WT for 25-52 weeks	Versus less than 5 weeks as baseline	-12.26	7.31	-26.60 – 2.07	0.094
Using WT for 53-78 weeks	Versus less than 5 weeks as baseline	-12.45	8.67	-29.44– 4.53	0.151
Using WT for 79-104 weeks	Versus less than 5 weeks as baseline	-3.54	7.37	-17.99 – 10.90	0.631
Using WT for more than 104 weeks	Versus less than 5 weeks as baseline	-3.32	8.8	-20.65 – 13.93	0.706
Water depth- CB	Versus dry belt as baseline	2.9	1.57	-0.17 – 5.97	0.064

WT exercise: Three times a month	Versus baseline fortnightly	-4.49	3.37	-11.09 – 2.11	0.182
WT exercise: Once a month	Versus baseline fortnightly	-9.76	5.27	-20.10 –0.57	0.064
WT exercise: Weekly	Versus baseline fortnightly	5.58	5.73	-5.64–16.81	0.33
Training depth- midcannon	Compared to MCPJ as baseline	9.5	5.64	-1.59– 20.52	0.094
Test speed	Per metre per second of speed	-0.56	2.73	-5.92– 4.79	0.837
Training speed	Per metre per second of speed	1.13	4	-6.71 – 8.97	0.778
Intercept	***Baseline craniocaudal ROM***	*46.28*	*0.48*		-
Using WT 5- 24 weeks	Versus less than 5 weeks as baseline	-4.45	*6.9*	-17.67 – 9.38	*0.548*
Using WT for 25-52 weeks	Versus less than 5 weeks as baseline	3.45	5.84	-8.00 – 14.91	0.554
Using WT for 53-78 weeks	Versus less than 5 weeks as baseline	-5.71	6.92	-19.27 – 7.83	0.408
Using WT for 79-104 weeks	Versus less than 5 weeks as baseline	4.58	5.89	-6.96 – 16.14	0.437
Using WT for more than 104 weeks	Versus less than 5 weeks as baseline	1.41	7.05	-12.40 – 15.23	0.841
Week 20	Compared to week 0	-0.31	0.71	-1.69 –1.07	0.655
WT exercise: Three times a month	Versus baseline fortnightly	-3.1	4.55	-12.03– 5.84	0.496
WT exercise: Once a month	Versus baseline fortnightly	4.6	4.21	-3.64 –12.86	0.274
Training depth- midcannon	Compared to MCPJ as baseline	-5.05	4.48	-13.80– 3.70	0.257
Training depth- Below carpus	Compared to MCPJ as baseline	-7.43	5.96	-19.16– 4.29	0.214
Training depth- Carpus	Compared to MCPJ as baseline	-4.7	4.93	-14.37– 4.97	0.341
Test speed	Per metre per second of speed	3.02	1.66	-0.22– 6.26	0.068
Training speed	Per metre per second of speed	5.46	3.19	-0.79 – 11.70	0.087
Sacrum		***(mm)***			
Intercept	***Baseline dorsoventral ROM***	*89.53*	*0.95*		-
Using WT for 25-52 weeks	Versus less than 5 weeks as baseline	-7.95	8.45	-24.51– 8.62	0.347
Using WT for 53-78 weeks	Versus less than 5 weeks as baseline	-6.79	10.01	-26.41– 12.82	0.497
Using WT for 79-104 weeks	Versus less than 5 weeks as baseline	-3.75	8.54	-20.50 – 12.98	0.660
Using WT for more than 104 weeks	Versus less than 5 weeks as baseline	-3.25	10.21	-23.27 – 16.76	0.750
Week 40	Compared to week 0	0.37	1.02	-1.62 –2.38	0.714
WT exercise: Three times a month	Versus baseline fortnightly	-6.28	6.6	-19.23– 6.64	0.342
WT exercise: Once a month	Versus baseline fortnightly	-8.87	6.1	-20.84 –3.10	0.146
Training depth- midcannon	Compared to MCPJ as baseline	9.79	6.39	-0.08– 8.69	0.126
Training depth- Below carpus	Compared to MCPJ as baseline	12.5	8.62	-4.40– 29.40	0.147
Training depth- Carpus	Compared to MCPJ as baseline	13.63	7.09	-0.28-27.54	0.055
Test speed	Per metre per second of speed	4.31	2.24	-0.09– 8.69	0.055
Training speed	Per metre per second of speed	7.31	4.62	-1.74 – 16.37	0.114
Intercept	***Baseline mediolateral ROM***	*66.84*	*0.89*		-
Using WT 5- 24 weeks	Versus less than 5 weeks as baseline	-6.82	*11.62*	-12.60 – 15.96	*0.557*
Using WT for 25-52 weeks	Versus less than 5 weeks as baseline	-0.54	9.82	-19.80 – 18.72	0.956
Using WT for 53-78 weeks	Versus less than 5 weeks as baseline	-6.12	11.63	-28.93 – 16.67	0.598
Using WT for 79-104 weeks	Versus less than 5 weeks as baseline	-1.89	9.93	-21.35– 17.57	0.849
Using WT for more than 104 weeks	Versus less than 5 weeks as baseline	15.87	11.87	-7.39 – 39.15	0.181
Week 20	Compared to week 0	-1.11	1.01	-3.08 –0.86	0.268
Week 40	Compared to week 0	-1.06	1.13	-3.28 –1.15	0.347

WT exercise: Three times a month	Versus baseline fortnightly	-0.89	7.67	-15.92 – 14.15	0.908
WT exercise: Once a month	Versus baseline fortnightly	-8.43	7.1	-22.35 –5.47	0.235
WT exercise: Weekly	Versus baseline fortnightly	-5.02	4.53	-13.90–3.86	0.268
Training depth- midcannon	Compared to MCPJ as baseline	0.47	7.43	-14.09– 15.04	0.949
Training depth- Below carpus	Compared to MCPJ as baseline	-2.26	10.02	-21.91– 17.37	0.821
Training depth- Carpus	Compared to MCPJ as baseline	3.26	8.25	-12.89– 19.42	0.692
Test speed	Compared to MCPJ as baseline	2.94	2.5	-1.95– 7.83	0.239
Training speed	Compared to MCPJ as baseline	3.2	5.37	-7.32– 13.73	0.551
Intercept	***Baseline craniocaudal ROM***	*47.86*	*0.58*		-
Using WT 5- 24 weeks	Versus less than 5 weeks as baseline	5.34	*9.24*	-12.77 – 23.48	*0.563*
Using WT for 25-52 weeks	Versus less than 5 weeks as baseline	2.84	7.82	-12.50 – 18.18	0.717
Using WT for 53-78 weeks	Versus less than 5 weeks as baseline	-3.04	9.25	-21.18 – 15.08	0.742
Using WT for 79-104 weeks	Versus less than 5 weeks as baseline	9.24	7.9	-6.23– 24.71	0.242
Using WT for more than 104 weeks	Versus less than 5 weeks as baseline	2.75	9.44	-15.76 – 21.26	0.771
Week 20	Compared to week 0	0.29	0.79	-1.27 –1.84	0.717
Week 40	Compared to week 0	-0.1	0.85	-1.76 –1.57	0.908
WT exercise: Three times a month	Versus baseline fortnightly	-4.96	6.08	-16.90 – 6.96	0.414
WT exercise: Once a month	Versus baseline fortnightly	5.34	5.63	-5.70 –16.39	0.343
WT exercise: Weekly	Versus baseline fortnightly	-5.28	3.6	-12.34–1.77	0.142
Training depth- midcannon	Compared to MCPJ as baseline	-5.92	5.95	-17.57– 5.75	0.32
Training depth- Below carpus	Compared to MCPJ as baseline	-7.32	7.99	-22.99– 8.34	0.359
Training depth- Carpus	Compared to MCPJ as baseline	-6.23	6.58	-19.12– 6.67	0.344
Test speed	Per metre per second of speed	2.41	1.91	-1.32– 6.16	0.206
Training speed	Per metre per second of speed	0.96	4.27	-7.39– 9.32	0.822
LTC		***(mm)***			
Intercept	***Baseline dorsoventral ROM***	*108.45*	*10.03*		-
Using WT for 25-52 weeks	Versus less than 5 weeks as baseline	-5.3	9.76	-24.44 – 13.87	0.587
Using WT for 53-78 weeks	Versus less than 5 weeks as baseline	-12.79	11.56	-35.46 – 9.87	0.269
Using WT for 79-104 weeks	Versus less than 5 weeks as baseline	-7.51	9.85	-26.81– 11.79	0.446
Using WT for more than 104 weeks	Versus less than 5 weeks as baseline	-1.9	11.78	-25.00– 21.20	0.872
Week 40	Compared to week 0	-0.95	0.85	-3.31 –1.41	0.4333
WT exercise: Three times a month	Versus baseline fortnightly	-0.48	7.61	-15.39– 14.43	0.95
WT exercise: Once a month	Versus baseline fortnightly	-6.2	7.05	-20.02 –7.60	0.378
Training depth- midcannon	Compared to MCPJ as baseline	6.48	7.45	-8.11– 21.07	0.384
Training depth- Below carpus	Compared to MCPJ as baseline	8.06	9.99	-11.53– 27.64	0.42
Training depth- Carpus	Compared to MCPJ as baseline	10.84	8.24	-5.30– 26.98	0.188
Training speed	Compared to MCPJ as baseline	8.11	5.34	-2.35– 18.58	0.129
Intercept	***Baseline mediolateral ROM***	*52.93*	*0.72*		-
Using WT 5- 24 weeks	Versus less than 5 weeks as baseline	-4.51	*8.59*	-21.34– 12.33	*0.6*
Using WT for 25-52 weeks	Versus less than 5 weeks as baseline	-3.26	7.27	-17.51– 10.99	0.654
Using WT for 53-78 weeks	Versus less than 5 weeks as baseline	2.68	8.61	-14.21 – 19.56	0.756
Using WT for 79-104 weeks	Versus less than 5 weeks as baseline	3.18	7.33	-11.20– 17.56	0.665

Using WT for more than 104 weeks	Versus less than 5 weeks as baseline	5.16	8.77	-12.04 – 22.35	0.557
Week 20	Compared to week 0	1.55	0.95	-1.27 –1.84	0.103
Week 40	Compared to week 0	1.5	0.95	-0.35–3.36	0.113
WT exercise: Three times a month	Versus baseline fortnightly	-10.87	5.67	-21.99– 0.24	0.055
WT exercise: Once a month	Versus baseline fortnightly	-6.8	5.25	-17.10 –3.48	0.195
WT exercise: Weekly	Versus baseline fortnightly	-6.54	3.35	-13.10–0.03	0.051
Training depth- midcannon	Compared to MCPJ as baseline	4.43	5.55	-6.44– 15.32	0.424
Training depth- Below carpus	Compared to MCPJ as baseline	9.69	7.45	-4.91– 24.28	0.193
Training depth- Carpus	Compared to MCPJ as baseline	2.68	6.14	-9.36– 14.71	0.663
Test speed	Per metre per second of speed	2.13	2.08	-1.94– 6.19	0.306
Training speed	Per metre per second of speed	0.5	3.98	-7.30– 8.29	0.901
Intercept	***Baseline craniocaudal ROM***	*57.6*	*0.59*		-
Using WT 5- 24 weeks	Versus less than 5 weeks as baseline	5.61	*9.01*	-12.03 – 23.27	*0.533*
Using WT for 25-52 weeks	Versus less than 5 weeks as baseline	-5.6	7.62	-20.54– 9.33	0.462
Using WT for 53-78 weeks	Versus less than 5 weeks as baseline	-4.96	9.02	-22.64 – 12.72	0.582
Using WT for 79-104 weeks	Versus less than 5 weeks as baseline	2.77	7.6	-12.30– 17.85	0.719
Using WT for more than 104 weeks	Versus less than 5 weeks as baseline	-1.43	9.2	-19.47 – 16.60	0.876
Week 20	Compared to week 0	-1.04	0.87	-2.73 –0.66	0.23
Water depth- PMC	Versus dry belt as baseline	1.25	1.04	-0.79 – 3.28	0.23
Water depth- DR	Versus dry belt as baseline	-0.78	1.04	-2.81– 1.25	0.451
WT exercise: Three times a month	Versus baseline fortnightly	-2.45	5.93	-14.08 – 9.17	0.679
WT exercise: Once a month	Versus baseline fortnightly	4.62	5.5	-6.15 –15.40	0.401
WT exercise: Weekly	Versus baseline fortnightly	-0.72	3.51	-7.59–6.16	0.838
Training depth- midcannon	Compared to MCPJ as baseline	-3.12	5.8	-14.50– 8.25	0.59
Training depth- Below carpus	Compared to MCPJ as baseline	4.56	7.79	-10.71– 19.83	0.559
Training depth- Carpus	Compared to MCPJ as baseline	-3.56	6.41	-16.14– 9.01	0.579
Test speed	Compared to MCPJ as baseline	3.44	1.93	-0.34– 7.22	0.075
Training speed	Per metre per second of speed	-4.15	4.17	-12.32– 4.01	0.319
RTC		***(mm)***			
Intercept	***Baseline dorsoventral ROM***	*110.14*	*1.0*		-
Using WT 5- 24 weeks	Versus less than 5 weeks as baseline	-24.32	*12.54*	-48.90 – 0.26	*0.563*
Using WT for 25-52 weeks	Versus less than 5 weeks as baseline	-5.24	10.62	-26.05– 15.56	0.717
Using WT for 53-78 weeks	Versus less than 5 weeks as baseline	-21.22	12.55	-45.82– 3.37	0.742
Using WT for 79-104 weeks	Versus less than 5 weeks as baseline	-9.97	10.71	-30.96– 11.01	0.242
Using WT for more than 104 weeks	Versus less than 5 weeks as baseline	-2.41	12.82	-27.54 – 22.70	0.771
Week 20	Compared to week 0	-1.86	0.97	-3.76 –0.49	0.056
Week 40	Compared to week 0	1.17	0.97	-3.07 –0.74	0.231
WT exercise: Three times a month	Versus baseline fortnightly	2.44	8.24	-13.91 – 18.60	0.767
WT exercise: Once a month	Versus baseline fortnightly	-0.27	7.64	-15.26 –14.72	0.972
Training depth- midcannon	Compared to MCPJ as baseline	3.76	8.05	-12.01– 19.55	0.64
Training depth- Below carpus	Compared to MCPJ as baseline	1.09	10.83	-20.13– 22.31	0.92
Training depth- Carpus	Compared to MCPJ as baseline	8.37	8.9	-9.08– 25.81	0.347

Test speed	Per metre per second of speed	1.8	2.21	-2.53– 6.13	0.416
Training speed	Per metre per second of speed	11.32	5.79	-0.03– 22.67	0.051
Intercept	***Baseline mediolateral ROM***	*56.53*	*0.8*		-
Using WT 5- 24 weeks	Versus less than 5 weeks as baseline	-3.71	*9.78*	-22.87 – 15.45	*0.705*
Using WT for 25-52 weeks	Versus less than 5 weeks as baseline	2.84	8.26	-13.35 – 19.02	0.731
Using WT for 53-78 weeks	Versus less than 5 weeks as baseline	2.44	9.81	-16.79 – 21.66	0.804
Using WT for 79-104 weeks	Versus less than 5 weeks as baseline	4.09	8.35	-12.29– 20.45	0.625
Using WT for more than 104 weeks	Versus less than 5 weeks as baseline	10.44	9.98	-9.11 – 30.00	0.295
WT exercise: Three times a month	Versus baseline fortnightly	-10.09	6.47	-22.78 – 2.56	0.119
WT exercise: Once a month	Versus baseline fortnightly	-8.49	5.99	-20.23 –3.25	0.156
Training depth- midcannon	Compared to MCPJ as baseline	0.23	6.26	-12.03– 12.50	0.97
Training depth- Below carpus	Compared to MCPJ as baseline	1.76	8.44	-14.76– 18.29	0.834
Training depth- Carpus	Compared to MCPJ as baseline	-1.84	6.93	-15.42– 11.74	0.791
Test speed	Per metre per second of speed	1.91	2.4	-2.80– 6.61	0.427
Training speed	Per metre per second of speed	1.72	4.53	-7.16– 10.59	0.705
Intercept	***Baseline craniocaudal ROM***	59.45	*0.54*		-
Using WT 5- 24 weeks	Versus less than 5 weeks as baseline	14.91	*8.21*	-1.19 – 31.00	*0.069*
Using WT for 25-52 weeks	Versus less than 5 weeks as baseline	8.02	6.93	-5.58 – 21.61	0.248
Using WT for 53-78 weeks	Versus less than 5 weeks as baseline	1.93	8.24	-14.22 – 18.07	0.815
Using WT for 79-104 weeks	Versus less than 5 weeks as baseline	7.19	7.02	-6.56– 20.94	0.305
Using WT for more than 104 weeks	Versus less than 5 weeks as baseline	0.14	8.38	-16.29 – 16.56	0.986
Week 20	Compared to week 0	-1.06	0.87	-2.76–0.63	0.217
Water depth- DR	Versus dry belt as baseline	1.42	1.09	-0.72– 3.56	0.194
WT exercise: Three times a month	Versus baseline fortnightly	-5.99	5.44	-16.65 – 4.66	0.27
WT exercise: Once a month	Versus baseline fortnightly	9.35	5.03	-0.51 –19.21	0.063
WT exercise: Weekly	Versus baseline fortnightly	1.41	3.21	-4.87–7.70	0.659
Training depth- midcannon	Compared to MCPJ as baseline	-5.08	5.26	-15.37– 5.22	0.334
Training depth- Below carpus	Compared to MCPJ as baseline	-9.82	7.09	-23.70– 4.07	0.166
Training depth- Carpus	Compared to MCPJ as baseline	-10.32	5.82	-21.72– 1.08	0.076
Test speed	Compared to MCPJ as baseline	0.38	2	-3.54– 4.31	0.849
Training speed	Per metre per second of speed	0.08	3.8	-7.38– 7.54	0.983

Table S3: Summary of non -significant predictors for flexion-extension and lateral bending range of motion during the water treadmill exercise test with the 10th, 13th and 18th thoracic vertebrae (T10, T13 and T18 respectively) and the 3rd and 5th lumbar vertebrae (L3 and L5 respectively) flexion-extension and lateral bending as outcome variables and test water depth and speed, week, prior treadmill experience, treadmill training in between tests (water depth, belt speed and training frequency), horses' level and discipline as predictors. Horse was included as a random factor to account for clustering due to repeated measures. CB = coronary band, MCPJ = metacarpophalangeal joint, PMC = proximal metacarpal region, DR = distal radius, midcannon = mid-proximal to distal metacarpus/metatarsus, WT = water treadmill, ROM = range of motion.

Outcome measure<br><br>**Predictor variable**	**Comparator for interpretation**	**Regression coefficient**	**Standard error**	**95% confidence interval of regression coefficient**	**P-value**
T10 ROM		(°)			
Flexion-extension					
Intercept	*Baseline flexion-extension ROM*	*8.81*	*0.1*		-
Using WT for 25-52 weeks	*Versus less than 5 weeks as baseline*	-0.97	0.81	-2.55 – 0.62	0.231
Using WT for 53-78 weeks	Versus less than 5 weeks as baseline	-1.42	0.97	-3.32 – 0.48	0.143
Using WT for 79-104 weeks	Versus less than 5 weeks as baseline	-1.03	0.82	-2.65 – 0.58	0.210
Using WT for more than 104 weeks	Versus less than 5 weeks as baseline	-1.16	0.98	-3.09 – 0.76	0.236
Week 20	Compared to week 0	0.14	0.16	-0.17 – 0.45	0.395
Week 40	Compared to week 0	-0.16	0.17	-0.49 – 0.17	0.343
WT exercise: Three times a month	Versus baseline fortnightly	-0.01	0.19	-0.33 – 1.31	0.988
WT exercise: Once a month	Versus baseline fortnightly	0.67	0.68	-0.19 – 0.54	0.279
Training depth- midcannon	Compared to MCPJ as baseline	0.1	0.62	-1.11 – 1.31	0.873
Training depth- Below carpus	Compared to MCPJ as baseline	0.16	0.85	-1.50 – 1.82	0.846
Training depth- Carpus	Compared to MCPJ as baseline	0.27	0.7	-1.11 – 1.64	0.704
Test speed	Per metre per second of speed	0.68	0.37	-0.05 – 1.41	0.068
Training speed	Per metre per second of speed	0.64	0.48	-0.29 – 1.57	0.178
Lateral bending					
Intercept	*Baseline lateral bending ROM*	*10.84*	*0.1*		-
Using WT for 5-24 weeks	Versus less than 5 weeks as baseline	-1.56	1.25	-4.01 – 0.89	0.212
Using WT for 25-52 weeks	Versus less than 5 weeks as baseline	-0.11	1.06	-2.18 – 1.96	0.918
Using WT for 53-78 weeks	Versus less than 5 weeks as baseline	-0.65	1.26	-3.11 – 1.82	0.606
Using WT for 79-104 weeks	Versus less than 5 weeks as baseline	-0.74	1.07	-2.84 – 1.37	0.494
Using WT for more than 104 weeks	Versus less than 5 weeks as baseline	-0.48	1.28	-2.98 – 2.02	0.707
Week 20	Compared to week 0	0.29	0.2	-0.10 – 0.45	0.151
Week 40	Compared to week 0	-0.11	0.2	-0.51 – 0.29	0.586
Water depth- PMC	Versus dry belt as baseline	-0.12	0.25	-0.61 – 0.36	0.632
WT exercise: Three times a month	Versus baseline fortnightly	-0.43	0.87	-2.12 – 1.27	0.622
WT exercise: Once a month	Versus baseline fortnightly	-0.57	0.89	-2.30 – 1.17	0.846
WT exercise: Weekly	Versus baseline fortnightly	-0.92	0.5	-1.89 – 0.05	0.063
Training depth- midcannon	Compared to MCPJ as baseline	-0.48	0.8	-2.04 – 1.08	0.549
Training depth- Below carpus	Compared to MCPJ as baseline	-0.17	1.09	-2.30 – 1.97	0.878
Training depth- Carpus	Compared to MCPJ as baseline	-0.3	0.9	-2.07– 1.46	0.74

Test speed	Per metre per second of speed	0.48	0.46	-0.42 – 1.40	0.298
Training speed	Per metre per second of speed	0.57	0.62	-0.66 – 1.79	0.055
T13 ROM		(°)			
Flexion-extension					
Intercept	*Baseline flexion-extension ROM*	*10.84*	*0.1*		
Using WT for 5-24 weeks	Versus less than 5 weeks as baseline	-1.63	1.03	-3.67 – 0.40	0.116
Using WT for 25-52 weeks	Versus less than 5 weeks as baseline	-0.5	0.87	-2.21 – 1.21	0.566
Using WT for 53-78 weeks	Versus less than 5 weeks as baseline	-1.27	1.04	-3.31 – 0.78	0.224
Using WT for 79-104 weeks	Versus less than 5 weeks as baseline	-1.1	0.88	-2.84 – 0.64	0.215
Using WT for more than 104 weeks	Versus less than 5 weeks as baseline	-0.55	1.06	-2.98 – 1.54	0.607
Week 20	Compared to week 0	-0.7	0.14	-0.33 – 0.20	0.609
Week 40	Compared to week 0	0.18	0.14	-0.98 – 0.46	0.206
WT exercise: Three times a month	Versus baseline fortnightly	-0.74	0.41	-1.55 – 0.50	0.066
WT exercise: Once a month	Versus baseline fortnightly	-0.43	0.66	-1.73 – 0.87	0.516
WT exercise: Weekly	Versus baseline fortnightly	-0.75	0.72	-1.30 – 1.53	0.873
Training depth- midcannon	Compared to MCPJ as baseline	0.24	0.67	-1.06 – 1.55	0.713
Training depth- Below carpus	Compared to MCPJ as baseline	0.41	0.91	-1.37 – 2.19	0.65
Training depth- Carpus	Compared to MCPJ as baseline	0.72	0.75	-0.74– 2.19	0.335
Test speed	Compared to MCPJ as baseline	0.23	0.34	-0.43 – 0.89	0.492
Training speed	Per metre per second of speed	0.48	0.51	-0.52 – 1.47	0.35
Lateral bending		(°)			
Intercept	*Baseline lateral bending ROM*	*9.23*	*0.14*		-
Using WT for 5-24 weeks	Versus less than 5 weeks as baseline	-0.83	1.18	-3.15 – 1.47	0.478
Using WT for 25-52 weeks	Versus less than 5 weeks as baseline	0.12	1	-1.82 – 2.07	0.902
Using WT for 53-78 weeks	Versus less than 5 weeks as baseline	-1.23	1.19	-3.55 – 1.09	0.299
Using WT for 79-104 weeks	Versus less than 5 weeks as baseline	-0.81	1.01	-2.79 – 1.17	0.424
Using WT for more than 104 weeks	Versus less than 5 weeks as baseline	-0.1	1.21	-2.46 – 2.26	0.935
Week 20	Compared to week 0	0.17	0.16	-0.15 – 0.50	0.289
Week 40	Compared to week 0	0.22	0.17	-0.11 – 0.56	0.183
WT exercise: Three times a month	Versus baseline fortnightly	-0.41	0.47	-1.48 – 1.70	0.066
WT exercise: Once a month	Versus baseline fortnightly	-0.02	0.84	-1.67 – 1.62	0.516
WT exercise: Weekly	Versus baseline fortnightly	0.11	0.81	-1.33 – 0.50	0.873
Training depth- midcannon	Compared to MCPJ as baseline	-0.05	0.75	-1.52 – 1.42	0.891
Training depth- Below carpus	Compared to MCPJ as baseline	-0.5	1.03	-1.32 – 0.50	0.376
Training depth- Carpus	Compared to MCPJ as baseline	0.34	0.85	-1.67– 1.63	0.98
Training speed	Per metre per second of speed	0.58	0.59	-0.58 – 1.73	0.327
T18 ROM		(°)			

Flexion-extension					
Intercept	*Baseline flexion-extension ROM*	*5.11*	*0.12*		-
Using WT for 5-24 weeks	Versus less than 5 weeks as baseline	-1.08	0.89	-2.83 – 0.66	0.221
Using WT for 25-52 weeks	Versus less than 5 weeks as baseline	0.1	0.74	-1.36 – 1.56	0.89
Using WT for 53-78 weeks	Versus less than 5 weeks as baseline	-1.21	0.9	-2.98 – 0.56	0.181
Using WT for 79-104 weeks	Versus less than 5 weeks as baseline	-0.04	0.77	-1.54 – 1.46	0.954
Using WT for more than 104 weeks	Versus less than 5 weeks as baseline	0.71	0.9	-1.05 – 2.47	0.431
Water depth- PMC	Versus dry belt as baseline	-0.19	0.32	-0.82 – 0.44	0.551
WT exercise: Three times a month	Versus baseline fortnightly	0.07	0.65	-1.20 – 1.34	0.917
WT exercise: Once a month	Versus baseline fortnightly	-0.03	0.36	-0.74 – 0.67	0.933
WT exercise: Weekly	Versus baseline fortnightly	0.24	0.58	-0.90 – 1.38	0.679
Training depth- midcannon	Compared to MCPJ as baseline	0.13	0.58	-1.00 – 1.27	0.713
Training depth- Below carpus	Compared to MCPJ as baseline	1.17	0.79	-0.38 – 2.71	0.65
Training depth- Carpus	Compared to MCPJ as baseline	1.06	0.67	-0.26– 2.37	0.335
Test speed	Per metre per second of speed	0.81	0.44	-0.06 – 1.67	0.068
Training speed	Per metre per second of speed	0.58	0.44	-0.28 – 1.44	0.189
Lateral bending		*(°)*			
Intercept	*Baseline lateral bending ROM*	*5.78*	*0.12*		-
Using WT for 25-52 weeks	Versus less than 5 weeks as baseline	-0.69	0.73	-2.12 – 0.73	0.342
Using WT for 79-104 weeks	Versus less than 5 weeks as baseline	-0.79	0.75	-2.25 – 0.67	0.290
Using WT for more than 104 weeks	Versus less than 5 weeks as baseline	0.53	0.62	-1.16 – 2.24	0.538
Water depth- Midcannon	Versus dry belt as baseline	0.15	0.32	-0.47 – 0.77	0.637
Water depth- Carpus	Versus dry belt as baseline	-0.19	0.32	-0.10 – 1.21	0.096
WT exercise: Three times a month	Versus baseline fortnightly	-0.15	0.61	-1.34 – 1.05	0.811
WT exercise: Once a month	Versus baseline fortnightly	-0.22	0.34	-0.89 – 0.45	0.516
WT exercise: Weekly	Versus baseline fortnightly	-0.003	0.58	-1.14 – 1.14	0.995
Test speed	Versus baseline fortnightly	0.23	0.43	-0.61 – 1.07	0.586
Training speed	Per metre per second of speed	0.41	0.42	-0.42 – 1.23	0.333
L3 ROM		*(°)*			
Flexion-extension					
Intercept	*Baseline flexion-extension ROM*	*7.61*	*0.11*		-
Using WT for 5-24 weeks	Versus less than 5 weeks as baseline	-1.04	0.94	-2.88– 0.80	0.268
Using WT for 25-52 weeks	Versus less than 5 weeks as baseline	-0.21	0.79	-1.75 – 1.33	0.792
Using WT for 53-78 weeks	Versus less than 5 weeks as baseline	-0.58	0.95	-2.44 – 1.28	0.541
Using WT for 79-104 weeks	Versus less than 5 weeks as baseline	-0.12	0.81	-1.70 – 1.46	0.882
Using WT for more than 104 weeks	Versus less than 5 weeks as baseline	-0.12	0.96	-2.00 – 1.76	0.898
Week 20	Compared to week 0	-0.24	0.16	-0.56 – 0.08	0.148
Week 40	Compared to week 0	0.43	0.17	-0.29 – 0.38	0.802
WT exercise: Three times a month	Versus baseline fortnightly	-0.69	0.37	-1.42 – 0.40	0.06
WT exercise: Once a month	Versus baseline fortnightly	0.55	0.61	-1.74 – 0.64	0.367

WT exercise: Weekly	Versus baseline fortnightly	0.39	0.59	-0.90 – 1.69	0.553
Training depth- midcannon	Compared to MCPJ as baseline	0.98	0.6	-0.21 – 2.17	0.106
Training depth- Below carpus	Compared to MCPJ as baseline	0.12	0.14	-1.50 – 1.74	0.886
Training depth- Carpus	Compared to MCPJ as baseline	1	1.46	-0.34– 2.35	0.143
Training speed	Per metre per second of speed	-0.3	0.146	-1.21 – 0.61	0.518
Lateral bending		*(°)*			
Intercept	*Baseline lateral bending ROM*	*8.05*	*0.12*		-
Using WT for 5-24 weeks	Versus less than 5 weeks as baseline	-1.3	1.13	-3.51 – 0.90	0.247
Using WT for 25-52 weeks	Versus less than 5 weeks as baseline	-0.32	0.95	-2.18 – 1.54	0.735
Using WT for 53-78 weeks	Versus less than 5 weeks as baseline	-0.38	1.13	-2.60 – 1.83	0.734
Using WT for 79-104 weeks	Versus less than 5 weeks as baseline	0.23	0.97	-1.65 – 2.12	0.810
Using WT for more than 104 weeks	Versus less than 5 weeks as baseline	-0.37	1.15	-2.63 – 1.88	0.745
Week 20	Compared to week 0	0.08	0.15	-0.21 – 0.37	0.575
Week 40	Compared to week 0	0.02	0.15	-0.28 – 0.32	0.909
Water depth- Midcannon	Versus dry belt as baseline	0.11	0.18	-0.24 – 0.46	0.528
Water depth- Carpus	Versus dry belt as baseline	0.05	0.2	-0.33 – 0.44	0.791
WT exercise: Three times a month	Versus baseline fortnightly	-0.77	0.45	-1.64 – 0.10	0.084
WT exercise: Once a month	Versus baseline fortnightly	-1.28	0.81	-2.87 – 0.29	0.109
WT exercise: Weekly	Versus baseline fortnightly	-0.04	0.78	-1.56 – 1.48	0.962
Training depth- Below carpus	Compared to MCPJ as baseline	0.45	0.46	-1.46 – 2.38	0.643
Training depth- Carpus	Compared to MCPJ as baseline	0.75	0.92	-0.83– 2.33	0.356
Test speed	Per metre per second of speed	-0.19	0.37	-0.91 – 0.53	0.612
Training speed	Per metre per second of speed	0.75	0.81	-0.84 – 1.59	0.387
L5 ROM		*(°)*			
Flexion-extension					
Intercept	*Baseline flexion-extension ROM*	*7.73*	*0.09*		-
Using WT for 5-24 weeks	Versus less than 5 weeks as baseline	-1.26	0.96	-3.13 – 0.61	0.187
Using WT for 25-52 weeks	Versus less than 5 weeks as baseline	-1.38	0.8	-2.96– 0.18	0.083
Using WT for 53-78 weeks	Versus less than 5 weeks as baseline	-1.23	0.96	-3.12 – 0.66	0.201
Using WT for 79-104 weeks	Versus less than 5 weeks as baseline	-0.67	0.82	-2.27 – 0.93	0.411
Using WT for more than 104 weeks	Versus less than 5 weeks as baseline	-1.41	0.98	-3.32 – 0.50	0.148
Week 20	Compared to week 0	0.04	0.17	-0.29 – 0.37	0.828
Week 40	Compared to week 0	0.2	0.18	-0.15 – 0.55	0.273
WT exercise: Three times a month	Versus baseline fortnightly	0.04	0.67	-1.26 – 0.23	0.177
WT exercise: Once a month	Versus baseline fortnightly	-0.51	-1.35	-1.90 – 0.52	0.265
WT exercise: Weekly	Versus baseline fortnightly	-0.69	-1.11	-1.28 – 1.36	0.951
Training depth- midcannon	Compared to MCPJ as baseline	0.82	0.62	-0.38 – 2.03	0.181
Training depth- Below carpus	Compared to MCPJ as baseline	0.35	0.84	-1.30 – 2.00	0.68
Training depth- Carpus	Compared to MCPJ as baseline	0.43	0.7	-0.94– 1.80	0.534
Test speed	Per metre per second of speed	0.74	0.39	-0.02 – 1.49	0.058
Training speed	Per metre per second of speed	-0.25	0.47	-1.17 – 0.68	0.6
Lateral bending		*(°)*			

Intercept	*Baseline lateral bending ROM*	*8.05*	*0.12*		-
Using WT for 5-24 weeks	Versus less than 5 weeks as baseline	-1.71	1.15	-3.98 – 0.56	0.14
Using WT for 25-52 weeks	Versus less than 5 weeks as baseline	-1.21	0.98	-3.12– 0.70	0.215
Using WT for 53-78 weeks	Versus less than 5 weeks as baseline	-1.12	1.16	-3.41– 1.15	0.333
Using WT for 79-104 weeks	Versus less than 5 weeks as baseline	-0.46	0.99	-2.40 – 1.49	0.646
Using WT for more than 104 weeks	Versus less than 5 weeks as baseline	-1.26	1.18	-3.57 – 1.06	0.289
Week 20	Compared to week 0	0.12	0.16	-0.19 – 0.42	0.446
Week 40	Compared to week 0	0.13	0.16	-0.18 – 0.44	0.426
WT exercise: Three times a month	Versus baseline fortnightly	-0.71	0.45	-1.61 – 0.18	0.084
WT exercise: Once a month	Versus baseline fortnightly	-1.78	0.83	-3.41– -0.16	0.109
WT exercise: Weekly	Versus baseline fortnightly	-0.46	0.8	-2.03 – 1.11	0.962
Training depth- Below carpus	Compared to MCPJ as baseline	0.72	1.01	-1.25 – 2.70	0.473
Training depth- Carpus	Compared to MCPJ as baseline	0.89	0.83	-0.74– 2.52	0.285
Test speed	Per metre per second of speed	-0.4	0.38	-1.14 – 0.37	0.302
Training speed	Per metre per second of speed	0.28	0.58	-0.86 – 1.41	0.631
